# Non-covalent assembly-enabled selectivity in aqueous microdroplets

**DOI:** 10.1039/d6sc00238b

**Published:** 2026-03-05

**Authors:** Zhiheng Ma, Pengju Wu, Xianlong Zhou, Lingchao Cai, Thomas Heine, Yu Jing

**Affiliations:** a Jiangsu Co-Innovation Centre of Efficient Processing and Utilization of Forest Resources, College of Chemical Engineering, Nanjing Forestry University Nanjing 210037 China xianlongzhou@njfu.edu.cn yujing@njfu.edu.cn; b TU Dresden, Fakultät für Chemie und Lebensmittelchemie Bergstraße 66c 01062 Dresden Germany thomas.heine@tu-dresden.de; c Center for Advanced Systems Unerstanding (CASUS), Helmholtz-Zentrum Dresden-Rossendorf Am Untermarkt 2 02826 Görlitz Germany; d Department of Chemistry, ibs Center for NanoMedicine, Yonsei University Seodaemun-gu Seoul 120-749 Republic of Korea

## Abstract

In microdroplets, various reactions are known to be accelerated. Yet, controlling chemoselectivity within droplets remains largely unexplored. Here, we show that non-covalent self-assembly in sprayed microdroplets enables selective, catalyst-free, ambient-temperature hydrogenation of multifunctional biomass-derived molecules. Using 5-hydroxymethylfurfural as a model system, we reveal that hydrogen bonding between its hydroxyl and aldehyde groups promotes supramolecular assembly, which selectively shields the carbonyl and hydroxyl moieties while exposing the furan ring to reduction. Spectroscopic measurements and density functional theory calculations confirm that this organization governs site-specific reactivity in the absence of external reductants or metal catalysts and amplify the electric field effect. Substrates lacking analogous hydrogen-bonding motifs undergo competing oxidation, underscoring the mechanistic role of molecular recognition. The strategy extends to furfural and furfuryl alcohol, demonstrating tunable product selectivity. These findings establish a general design principle in which confined microenvironments and supramolecular assemblies cooperate to direct chemoselectivity, offering a sustainable approach to selective transformations beyond conventional catalysis.

## Abstract 2

Microdroplets enable selective, catalyst-free hydrogenation of biomass molecules *via* self-assembly and hydrogen bonding, paving the way for sustainable, tunable transformations.

## Graphical abstract

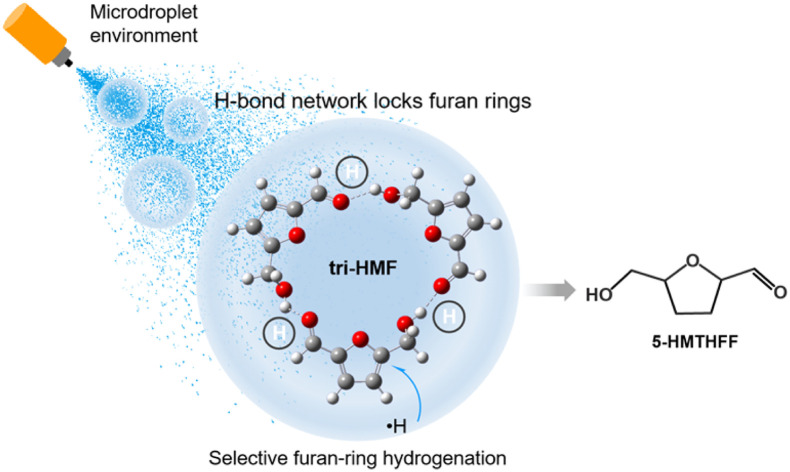

## Introduction

Microdroplet chemistry has recently emerged as a powerful tool for promoting chemical transformations under mild conditions, without added catalysts, applied voltages, or elevated temperatures.^1–4^ The intense electric fields and high surface-to-volume ratios at droplet interfaces can drive both oxidation and reduction reactions with dramatically enhanced kinetics.^5–7^ Functioning as self-contained redox systems,^8^ microdroplets generate electrons and reactive species such as ˙OH and H_2_O_2_,^9,10^ enabling cascade transformations of complex molecules including CO_2_,^11–15^ NH_3_,^16–18^ N_2_,^19–21^ propylene^3^ and *etc.* Yet this dual redox character introduces a fundamental uncertainty: within multifunctional substrates, will hydrogenation dominate, or will oxidation prevail? To date, most studies emphasize rate acceleration, while the capacity to impose selective control within microdroplets remains largely unexplored. Unlocking this capability would transform microdroplets from a kinetic curiosity into a programmable platform for sustainable synthesis.

**Scheme 1:**
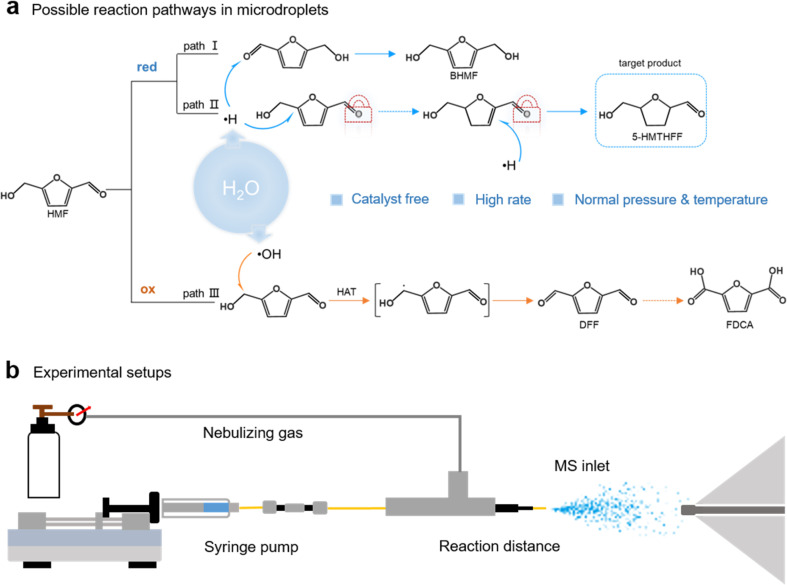
Schematic illustration of (a) possible reaction pathways for HMF conversion under microdroplet conditions. Both reduction and oxidation of HMF are possible due to the coexistence of ˙H and ˙OH, giving rise to a variety of competing products, including 2,5-bis-(hydroxymethyl) furan (BHMF), 2,5-bis (hydroxymethyl) tetrahy-drofuran (BHMTHF), 5-HMTHFF, diformylfuran (DFF) and 2,5-furandicarboxylic acid (FDCA). Because aldehyde hydrogenation is typically more favorable than furan-ring hydrogenation, achieving selective reduction of the furan ring requires locking the aldehyde group as illustrated in the scheme; (b) the experimental setup for the generation and detection of HMF microdroplets.

This question is particularly relevant for biomass-derived platform molecules such as 5-hydroxymethylfurfural (HMF) and furfural (FAL), whose multifunctional structures provide rich opportunities for valorization but also competing reactivities.^22–24^ In HMF, hydrogenation may occur at the aldehyde group, the furan ring, or both, while oxidation typically first targets the alcohol group. Each pathway produces distinct products with specific applications (Scheme 1a).^25–28^ Among these, selective furan-ring hydrogenation affords 5-hydroxymethyltetrahydrofurfuryl alcohol (5-HMTHFF),^29,30^ a high-value precursor for pharmaceuticals including ranitidine, furosemide, and cefuroxime.^31^ Achieving this transformation requires inverting conventional selectivity trends by suppressing aldehyde reduction, which is otherwise the preferred pathway.^32^ Conventional catalytic methods rely on noble metals, pressurized hydrogen, and elevated temperatures,^28,29^ while electrocatalytic strategies remain catalyst-dependent and often non-selective.^33–36^ Thus, a catalyst-free, ambient-temperature method to enforce furan-selective hydrogenation would be highly desirable.

Here, we demonstrate that non-covalent self-assembly within aqueous microdroplets enables catalyst-free hydrogenation of HMF to 5-HMTHFF under ambient conditions with complete furan selectivity. Mechanistic analyses combining nuclear magnetic resonance spectroscopy (NMR), mass spectrometry, density functional theory (DFT), and *ab initio* molecular dynamics reveal that hydrogen bonding between the hydroxyl and aldehyde groups of HMF drives supramolecular dimer and trimer formation. These assemblies selectively shield carbonyl sites while exposing the furan ring to reduction at the droplet interface. In contrast, analogs lacking dual hydrogen-bonding capacity undergo oxidation rather than hydrogenation, forming 2(5*H*)-furanone (FRO) and fumaric acid (FmA), which highlights the decisive role of molecular recognition. This strategy extends to furfural and furfuryl alcohol (FOL), offering tunable oxidation or reduction outcomes depending on functional group composition and droplet conditions. Together, these findings establish that supramolecular organization and confined microenvironments can act cooperatively to dictate selectivity, advancing microdroplet chemistry from a phenomenon of rate acceleration to a general design principle for selective, sustainable transformations.

## Results and discussion

### Conversion of HMF under microdroplet conditions

**Fig. 1:**
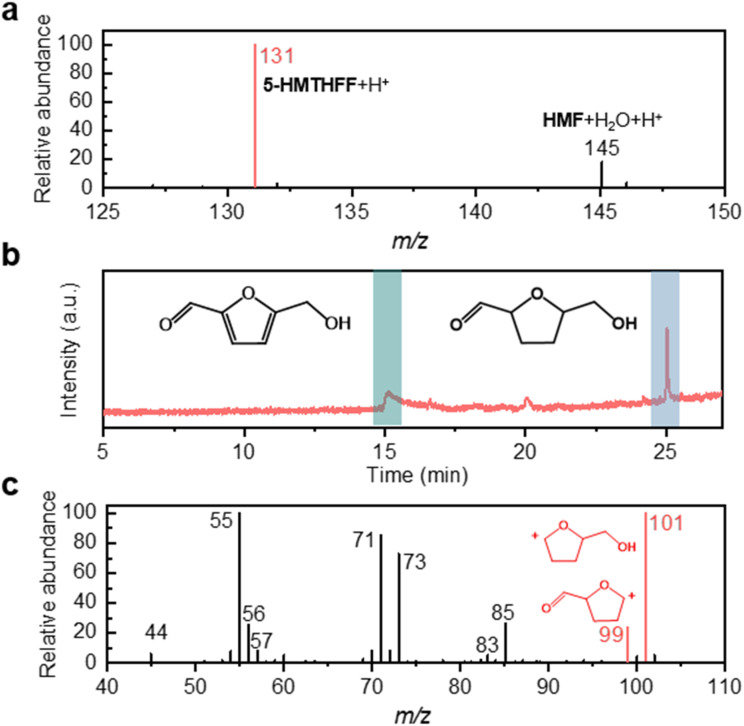
Characterization of HMF conversion in microdroplets. (a) Mass spectrum in positive mode of HMF microdroplets; (b) GC-MS chromatogram of collected HMF microdroplets containing the produced 5-HMTHFF; (c) GC-MS mass spectrum of chromatogram at the 25 min retention time.

To understand the conversion of HMF under microdroplet conditions, microdroplets were generated by adiabatic expansion of aqueous HMF solution (2–20 mmol L^−1^) using the setup shown in Scheme 1b,^37–41^ and subsequently analyzed *via* MS or collected for gas chromatography-mass spectrometry (GC-MS) and NMR characterization (see Methods for details). The mass spectrum (Fig. 1a) exhibits a peak at *m*/*z* 145, which is assigned to the [HMF + H_2_O + H]^+^ adduct. Second-stage mass spectrometry (MS^2^) analysis confirmed its identity by yielding a *m*/*z* 127 fragment ([HMF + H]^+^, Fig. S1), which is formed through collision-induced dissociation (CID) of the [HMF + H_2_O + H]^+^ adduct. A distinct peak observed at *m*/*z* 131 corresponds to 5-HMTHFF, indicating selective hydrogenation of HMF's furan ring. GC-MS analysis of collected microdroplets confirm the identity of the generated product (the microdroplet collection setup is shown in Fig. S2). As shown in Fig. 1b, chromatographic analysis revealed two distinct peaks at retention times of around 15 min and 25 min, corresponding to the reactant HMF (Fig. S3) and the product 5-HMTHFF (Fig. 1c), respectively. The latter peak exhibits characteristic fragment ions at *m*/*z* 99 and 101, which are absent in bulk-phase HMF solution (Fig. S4 and 5), consistent with reported fragmentation patterns of 5-HMTHFF.^42,43^ Specifically, the *m*/*z* 99 peak is attributed to the dehydroxylated 5-HMTHFF fragment, while the *m*/*z* 101 peak arises from the cleavage of the aldehyde moiety.

By modulating the reaction conditions, we next investigated the experimental parameters that affecting the yield of 5-HMTHFF (Fig. S6). By elevating the sheath gas pressure from 80 to 160 psi (Fig. S6a), we observed a moderate increase in product intensity. Previous studies have demonstrated that increasing the sheath gas pressure reduces droplet size,^44–46^ thereby increasing the droplet surface area-to-volume ratio and improving the product conversion efficiency. To probe the structure–activity relationship, we investigated the effect of sheath gas pressure and flow rate on ˙H radical intensity using TEMPO as a radical scavenger (Fig. S7). The experimental results show that the absolute intensity of ˙H increases with increasing sheath gas pressure (Fig. S7a). This suggests that the pressure-induced reduction in droplet size increases the surface area-to-volume ratio, thereby promoting ˙H generation and enhancing the yield of the target product (Fig. S8a). Similarly, increasing the flow rate (from 5 to 25 µL min^−1^) by raising the syringe pump pushing speed results in higher ˙H intensity (Fig. S7b). This is because a higher shear force at the needle tip promotes the formation of smaller microdroplets. However, despite the increase in ˙H intensity, the product yield decreases under higher flow rates (Fig. S6b), owing to insufficient reaction residence time. Compared with the substantial increase in ˙H concentration achieved by elevating the pressure, the enhancement induced by increasing the flow rate is relatively modest and cannot compensate for the shortened reaction time (Fig. S8b). The critical role of reaction residence time was particularly evident in the regulation of reaction distance, which underscores the dual importance of droplet size and residence time in the reaction system. The yield of 5-HMTHFF could be effectively tuned by varying the spray distance: extending the spray distance from 5 mm to 25 mm elevated the product yield from 85% to 97% (Fig. S6c). Finally, increasing the HMF concentration from 2 mmol L^−1^ to 20 mmol L^−1^ also effectively improved the product yield from 85% to 98% (Fig. S6d).

To explore the synergistic effects between different reaction parameters, we designed five experimental groups with varying sheath gas pressures, flow rates, spray distances and HMF concentrations to evaluate their combined impacts on product yield (Fig. S9). As discussed earlier, the limited yield enhancement at high pressures is ascribed to shortened spray duration. Therefore, a longer spray distance under high-pressure conditions was adopted to extend the reaction residence time. Meanwhile, a lower flow rate and higher HMF concentration were employed to further prolong the reaction time. Under these optimized combined conditions, the yield of 5-HMTHFF increased from 81% to 98%. In summary, increasing the sheath gas pressure reduces droplet size and increases the surface area-to-volume ratio of the droplets, thereby strengthening interfacial effects. In contrast, decreasing the flow rate and extending the spray distance prolong the reaction residence time, allowing more complete transformation. By tuning these parameters to tailor the physicochemical properties of microdroplets, the reaction efficiency can be effectively enhanced.

Compared with traditional catalytic methods for the hydrogenation of HMF to 5-HMTHFF, the present protocol proceeds under catalyst-free, pressure-free and ambient-temperature conditions (Table S3). Hydrogen radicals generated spontaneously in microdroplets serve as the hydrogen source (Fig. S10), obviating the need for external H_2_ gas. Given the difficulty of selectively producing 5-HMTHFF from HMF using conventional catalytic methods, its exclusive formation under ambient microdroplet conditions underscores the efficiency of this approach.

Radical-trapping experiments with TEMPO revealed the coexistence of ˙H and ˙OH radicals in HMF solution (characteristic peaks at *m*/*z* 157 ([TEMPO + ˙H]^+^) and *m*/*z* 174 ([TEMPO + ˙OH]^+^) in Fig. S10). Furthermore, trapping experiments of free radicals generated from pure water nebulization revealed a distinct characteristic peak corresponding to TEMPO-H at *m*/*z* 157 (Fig. S11). This observation confirms that water serves as the hydrogen source for HMF hydrogenation (Fig. S12). The underlying mechanism originates from the strong electric field present at the air–water interface of microdroplets. Under this intense interfacial electric field, water molecules undergo homolytic cleavage to generate ˙H and ˙OH radicals.^9,19^ The resulting ˙H radicals act as direct hydrogen donors in the hydrogenation process. Surprisingly, despite this coexistence, furan-ring hydrogenation proceeds selectively without concurrent oxidation or reduction of the aldehyde or hydroxymethyl groups. Moreover, increasing the TEMPO concentration suppressed 5-HMTHFF formation (Fig. S13), indicating that ˙H radicals in the droplets drive this transformation. These findings point to a unique mechanistic pathway in which furan-ring hydrogenation is selectively promoted while side-chain reduction or oxidation is suppressed, warranting further mechanistic investigation.

### Hydrogen bonding-guided selectivity in HMF hydrogenation

**Fig. 2:**
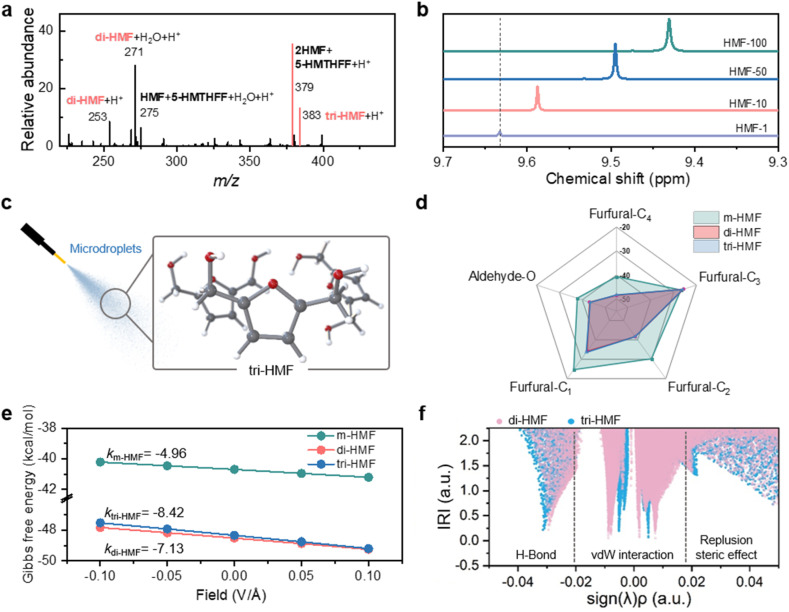
Mechanism for selective HMF conversion in microdroplets. (a) Positive mass spectrum of HMF with high mass-to-charge ratio; (b) ^1^H NMR peak shifts of the aldehyde proton in HMF as a function of HMF concentration (1, 10, 50, 100 µL in CDCl_3_); (c) schematics for microdroplet spraying containing HMF oligomers; (d) comparison of hydrogenation energies at different binding sites of HMF between monomer, dimer and trimer; (e) hydrogenation energy of the 4th carbon of HFM in the monomer, dimer and trimer as a function of the electric field; (f) IRI analysis of di-HMF and tri-HMF. Regions where sign(*λ*)*ρ* < −0.02 correspond to contribution of hydrogen bonding (H-bond), the range of −0.02 < sign(*λ*)*ρ* < 0.02 indicates van der Waals (vdW) dominated contributions. The sign(*λ*) function serves as a symbolic indicator, taking values of −1 for *λ* < 0 and +1 for *λ* > 0 (the *λ* < 0 and *λ* > 0 sub-tables represent regions where the electron density accumulates and depletes along specific directions, respectively). The strength of the weak interaction force is determined by *ρ*, that is, the greater |*ρ*|, the stronger the force corresponding to sign(*λ*) *ρ*.

To understand the selective hydrogenation mechanism in microdroplets, we examined the high *m*/*z* region of the mass spectra. As shown in Fig. 2a, peaks at *m*/*z* 253 and 271 correspond to a non-covalent HMF dimer (di-HMF) and their water adduct ([di-HMF + H_2_O + H]^+^), while the *m*/*z* 379 peak indicates a HMF trimer (tri-HMF) species. The detection of mixed oligomers containing both HMF and 5-HMTHFF (*m*/*z* 275 and 383 in Fig. 2a) suggests partial hydrogenation within oligomeric assemblies. Specifically, the peak at *m*/*z* 275 corresponds to one HMF molecule combined with one 5-HMTHFF, while the peak at *m*/*z* 383 is attributed to two HMF molecules with one 5-HMTHFF. These observations underscore the significance of intermolecular interactions in HMF solutions, which can be understood as the aldehyde and hydroxyl groups at the termini of HMF form reciprocal hydrogen bonds, two in the dimer and three in the trimer.^47,48^ This hydrogen bonding likely shields the terminal functional groups from redox activity while leaving the furan ring accessible for selective hydrogenation. Supporting this, variable-concentration ^1^H NMR spectra (Fig. 2b) show an upfield shift of the aldehyde proton with increasing HMF concentration, indicating enhanced hydrogen bonding (Fig. S14).

DFT calculations (Table S1) further reveal that the hydrogen bonding interactions are energetically favorable, with binding energies of −9.9 and −15.4 kcal mol^−1^ for di-HMF and tri-HMF, respectively, stronger than that between HMF and water (Fig. S15). Optimized structures exhibit typical hydrogen bonds of ∼1.85 Å linking aldehyde and hydroxyl groups in ring-like configurations (Fig. 2c), which are stable at the air–gas surface as demonstrated by AIMD simulations (Fig. S16 and S17). We then examined the initial hydrogenation of HMF in its dimeric and trimeric forms, in comparison with the monomer (Table S2). Five potential hydrogenation sites were considered, one at the aldehyde oxygen and four at the carbon atoms of furan ring (Fig. 2c). As illustrated in Fig. 2d, the hydrogenation energies at all active sites of HFM significantly decrease upon oligomerization of HMF. Notably, the hydrogenation energy (−40.69 kcal mol^−1^) at the 4th carbon of the furan ring, initially comparable to that of the aldehyde oxygen (−37.82 kcal mol^−1^) in the monomer, becomes markedly more negative in both the di-HMF (−48.52 kcal mol^−1^) and tri-HMF (−48.34 kcal mol^−1^). The 4th carbon becomes the most reactive site for hydrogenation in the oligomers.

Recognizing the interfacial electric fields in microdroplets,^49,50^ we examined their effect on the hydrogenation of the furan carbon. As shown in Fig. 2e, external electric fields (∼10^9^ V m^−1^, reported based on both experimental and computational grounds as measured by stimulated Raman excited fluorescence microscopy spectroscopy and confirmed by coarse-grained dynamics simulations^4^) promote the hydrogenation of HMF in all forms. Notably, the promoting effect is substantially stronger for di-HMF and tri-HMF. The slope of the hydrogenation energy as a function of electric field is significantly smaller for monomeric HMF than for dimeric and trimeric species. This behavior arises from hydrogen-bond-mediated charge delocalization in oligomers (Fig. S18), which amplifies the polarization response under an external electric field. The hydrogenation energies and field-dependent trends for dimer and trimer are nearly identical, implying a comparable influence on their selective hydrogenation in microdroplets. This similarity arises from the analogous ring-like configurations and equivalent hydrogen bonding effects in dimer and trimer. Specifically, di-HMF and tri-HMF exhibit similar frontier orbitals (Fig. S18), and the hydrogen bonding energy per unit is consistent at approximately −5.07 kcal mol^−1^ per unit. The interaction region indicator (IRI) analysis performed using Multiwfn (Fig. 2f),^51–53^ provides an intuitive visualization of the regions and types of intermolecular interactions, further confirming that hydrogen bonding contributes equivalently to the intermolecular interactions in both di-HMF and tri-HMF.

These computational results conclusively identify hydrogen bonding as the primary factor shielding the aldehyde and hydroxyl groups of HMF from redox reactions in microdroplets. Meanwhile, these interactions amplify the electric field effect, thereby promoting the selective hydrogenation of the furan ring and facilitating the formation of 5-HMTHFF.

### Disruption of hydrogen bonding alters the reactivity of HMF analogs in microdroplets

**Fig. 3:**
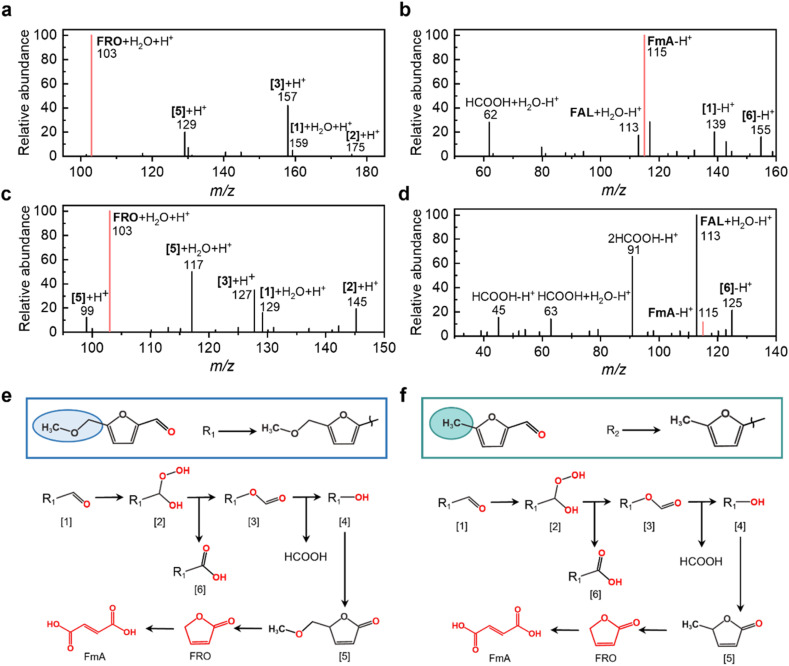
Conversion of 5-methoxymethyl-2-furaldehyde and 5-methylfurfural in microdroplets. (a) Positive and (b) negative mode mass spectra of microdroplet spray of 5-methoxymethyl-2-furaldehyde solution; (c) positive and (d) negative mode mass spectra of microdroplet spray of 5-methylfurfural solution; schematics for the possible reaction pathway during the oxidation of (e) 5-methoxymethyl-2-furaldehyde and (f) 5-methylfurfural in microdroplets.

To further validate the pivotal role of hydrogen bonding in dictating HMF's selective reactivity, we examined the reactivity of two structural analogs, *i.e.*, 5-methoxymethyl-2-furaldehyde and 5-methylfurfural, under identical conditions. These two analogs can be regarded as HMF derivatives obtained through functionalization at the 5-position, either by methylation of the hydroxymethyl group or by complete substitution with a methyl group. In this way, the original intermolecular hydrogen bonding interactions presented in HMF are disrupted.^54^ As demonstrated in Fig. 3a and b, 5-methoxymethyl-2-furaldehyde undergoes oxidation instead of selective hydrogenation at the furan ring, leading to the formation of FRO and FmA, as evidenced by peaks at *m*/*z* 103 (positive mode) and *m*/*z* 115 (negative mode), respectively. Similarly, 5-methylfurfural is also converted into the oxidized products, as confirmed by its mass spectra (Fig. 3c and d).

These observations are consistent with the oxidative environment of microdroplets, which contain reactive oxygen species such as ˙OH radicals and H_2_O_2_.^9^ In the absence of protective intermolecular hydrogen bonding, the aldehyde group of 5-methoxymethyl-2-furaldehyde becomes susceptible to nucleophilic attack by oxidants, leading to the formation of unstable intermediates. For 5-methoxymethyl-2-furaldehyde, this leads to the formation of peroxy-5-methoxymethyl-2-furaldehyde ([2], Fig. 3e) as evidenced by the peak at *m*/*z* 175 in Fig. 3a. Notably, the acidic environment of microdroplets promotes this nucleophilic addition by protonating the aldehyde group (evidenced by peaks at *m*/*z* 159, 139 in Fig. 3a and b), thereby enhancing the electrophilicity of the carbonyl carbon and facilitating the formation of the peroxy adduct. The intermediate [2] is converted into 5-(methoxymethyl) furfural formate ([3]) and 5-(methoxymethyl)-2-furoic acid ([6]) through Baeyer–Villiger oxidation, as indicated by peaks at *m*/*z* 157, 155. Intermediate [3] is subsequently hydrolyzed to 5-(methoxymethyl) furan-2-ol ([4]), which readily undergoes keto-enol tautomerization to form 5-(methoxymethyl) furan-2-carbaldehyde ([5], *m*/*z* 129). This transient complex is inherent unstable that induces cleavage of the methoxymethyl moiety, leading to the formation of FRO (*m*/*z* 103). Finally, the FRO undergoes oxidative ring-opening to form FmA (evidenced by the peak at *m*/*z* 115 in Fig. 3b). Fig. 3e displays the full reaction cascade.^55^ As shown in Fig. 3f, 5-methylfurfural in microdroplets follows a similar reaction pathway towards the formation of FRO and FmA.

The above results confirm the essential role of intermolecular hydrogen bonding in steering HMF's reactivity. In HMF, strong intermolecular hydrogen bonding (two hydrogen bonds in the dimer and three in the trimer), stabilizes the hydroxyl and aldehyde groups, shielding them from oxidative pathways and directing the reaction toward selective hydrogenation of the furan ring. In contrast, the absence of such cooperative hydrogen bonding in 5-methoxymethyl-2-furaldehyde and 5-methylfurfural permits oxidative transformations. One might question why hydrogen bonding with water does not confer similar protection for the aldehyde group of 5-methoxymethyl-2-furaldehyde and 5-methylfurfural from oxidation. The answer lies in the relative weakness and lack of structural enforcement of single H-bonds with water, as opposed to the robust, sterically protective ring-like networks formed in HMF oligomers. These structural features not only stabilize reactive groups but also restrict access to oxidative species, uniquely enabling the selective hydrogenation observed for HMF in microdroplets.

### Upgradation of FAL and FOL in microdroplets

**Fig. 4:**
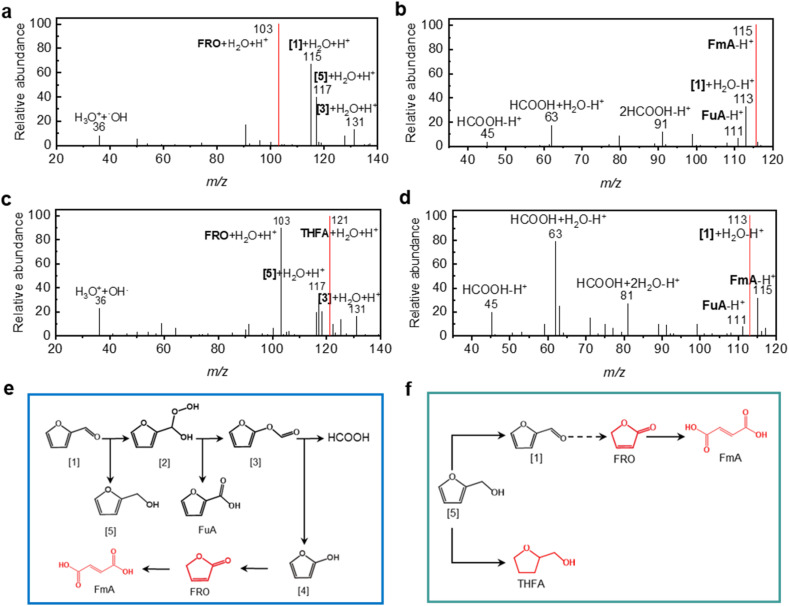
FAL & FOL conversion. (a) Positive and (b) negative mode mass spectra of microdroplet spray of FAL solution; (c) positive and (d) negative mode mass spectrum of microdroplet spray of FOL solution; schematics for the reaction pathway of FAL (e) and (f) FOL conversion in microdroplets.

Building on the demonstrated role of intermolecular hydrogen bonding in facilitating selective conversion of HMF to 5-HMTHFF, we examined the transformation of other biomass-derived furan compounds, including FAL and FOL under identical microdroplet conditions. FAL is a widely recognized platform chemical often co-produced with HMF during the industrial processing of lignocellulosic biomass. FOL, on the other hand, is a typical hydrogenated derivative of FAL. Structurally, both FAL and FOL can be regarded as simplified analogs of HMF, retaining only a single functional group, an aldehyde in FAL or a hydroxyl in FOL. By nebulizing aqueous solution of FAL and FOL through a syringe, we generated and analyzed their respective microdroplets (Fig. 4a–d). Similar to the cases of 5-methoxymethyl-2-furaldehyde and 5-methylfurfural, FAL undergoes oxidation in microdroplets due to the presence of ˙OH radicals (Fig. S19), leading to the formation of FRO and FmA, as confirmed in Fig. 4a and b. The proposed reaction pathway (Fig. 4e), begins with nucleophilic addition of H_2_O_2_ to the aldehyde group of FAL, forming a peroxy intermediate ([2]). Through a Baeyer–Villiger oxidation, this intermediate is converted into furfural formate ([3], *m*/*z* 131) and 2-Furoic acid (FuA, *m*/*z* 111). Subsequent hydrolysis of furfural formate produces furan-2-ol ([4]) and formic acid (*m*/*z* 45). The unstable furan-2-ol rapidly tautomerizes to generate FRO in the hydrated form, [FRO + H_2_O + H]^+^, as evidenced by the peak at *m*/*z* 103. This assignment is supported by MS^2^ analysis, in which the parent ion at *m*/*z* 103 undergoes CID to yield a fragment at *m*/*z* 85 (Fig. S20), corresponding to the loss of H_2_O and the formation of FRO. Finally, FRO undergoes ring-opening to generate FmA (*m*/*z* 115), whose identity is validated by MS^2^ fragmentation to *m*/*z* 71 ([C_3_H_4_O_2_–H]^−^, Fig. S21) and confirmed by ^1^H NMR spectroscopy (Fig. S22). In addition to these key species, hydrated adducts at *m*/*z* 115 ([FAL + H_2_O + H]^+^), 117 ([FOL + H_2_O + H]^+^), and 131 ([2-formyloxyfuran + H_2_O + H]^+^) were detected and confirmed by MS^2^ analysis (Fig. S23–S25), substantiating the proposed intermediates.

In the absence of intermolecular hydrogen bonding, FOL exhibits similar oxidative reactivity in microdroplets (Fig. 4c and d). The resulting oxidation products closely resemble those of FAL. Specifically, FOL is initially oxidized to FAL (as indicated by the peak at *m*/*z* 113) in the present of ˙OH radicals (Fig. S26) and subsequently follows the same oxidation pathway (Fig. 4f) to yield FRO (*m*/*z* 103) and FmA (*m*/*z* 115). These assignments are corroborated by MS^2^ analysis (Fig. S27–S29). Interestingly, FOL also undergoes hydrogenation of the furan ring, producing tetrahydrofurfuryl alcohol (THFA), as evidenced by the peak at *m*/*z* 121 in Fig. 4c. This indicates that under microdroplet conditions, FOL is susceptible to both oxidative and reductive transformations. This is because only substrates bearing bifunctional groups capable of forming intermolecular hydrogen bonds, can undergo selective transformation in microdroplets. In contrast, non-aromatic substrates or aromatic substrates lacking cooperative hydrogen-bonding motifs do not exhibit selective hydrogenation. Thus, aromatic compounds such as HMF, which possess both aldehyde and hydroxyl groups and can form synergistic hydrogen bonding networks, are uniquely suited for selective furan-ring hydrogenation. These findings highlight the significant influence of functional group composition on the reactivity of furanic compounds in microdroplets. While both FAL and FOL are oxidized in the absence of stabilizing hydrogen bonding networks, only compounds like HMF, with both aldehyde and hydroxyl groups enabling cooperative hydrogen bonding, undergo selective furan ring hydrogenation. This underscores the unique reactivity of HMF and the pivotal role of supramolecular interactions in directing microdroplet chemistry.

Notably, FRO is a valuable intermediate in organic synthesis and serves as both a wood plasticizer and preservative. FmA, meanwhile, has broad applications across the chemical, agricultural, and pharmaceutical industries.^56,57^ Therefore, microdroplet chemistry offers a green and sustainable strategy for upgrading biomass-derived platform molecules into value-added products. Further optimization experiments reveal that low concentration, low flow rate, low pressure, and extended reaction distance favor the formation of FRO from FAL (Fig. S30). By contrast, the yield of FmA increases under conditions of high concentration, high flow rate, elevated pressure, and longer reaction distance. Moreover, introducing molecular oxygen into the N_2_ sheath gas enhances FRO production (Fig. S31), as O_2_ reacts with ˙H radicals to generate the highly oxidative ˙OOH species,^58,59^ thereby promoting FAL oxidation. For FOL, the production of THFA is favored under conditions of higher gas pressure, reduced flow velocity, extended reaction distance and increased concentration of the reactant, as evidenced by the increased yield under these conditions (Fig. S32).

## Conclusions

We have demonstrated the potential of microdroplet chemistry as a green, rapid, and catalyst-free strategy for selectively upgrading biomass-derived furanic molecules into high-value chemicals. Through a combined experimental and theoretical approach, we revealed the pivotal role of intermolecular hydrogen bonding between the aldehyde and hydroxyl functional groups in HMF. These interactions protect HMF from oxidation and direct the selective hydrogenation of the furan ring, ultimately yielding the valuable product 5-HMTHFF. In contrast, HMF analogs including 5-methoxymethyl-2-furaldehyde and 5-methylfurfural, which lack the capacity for such hydrogen bonding, are readily oxidized in the presence of redox species within microdroplets. We extended this strategy to other biomass platform molecules including FAL and FOL. By tuning reaction parameters, including concentration, flow rate, pressure, and reaction distance, we successfully modulated product selectivity to obtain FRO and FmA from FAL, and THFA from FOL. Overall, our findings establish microdroplet chemistry as a versatile and sustainable approach for biomass valorization, while offering new insight into controlling chemoselectivity through intermolecular interactions.

## Author contributions

Z. M., X. Z., and Y. J. conceived and designed this work. Z. M. was responsible for the conduction of the experiments and products characterization. P. W. performed the calculations. Z. M., P. W., X. Z., T. H. L. C. and Y. J. contributed to the data analysis. Z. M., X. Z. T. H. and Y. J. wrote the paper together. X. Z. and Y. J. co-directed the research.

## Conflicts of interest

The authors declare no competing financial interest.

## Supplementary Material

SC-017-D6SC00238B-s001

## Data Availability

The data supporting this article have been included as part of the supplementary information (SI). Supplementary information: experiment details, experimental and computational methods, additional results on MS experiments and DFT calculations. See DOI: https://doi.org/10.1039/d6sc00238b. The computational data that support the findings of this study are openly available in NOMAD at https://dx.doi.org/10.17172/NOMAD/2026.01.08-2. Structure information of HMF monomer, dimer and trimer can be found in NOMAD with DOI: 10.17172/NOMAD/2026.01.08-2.
