# Supplementary material for: Non-covalent assembly-enabled selectivity in aqueous microdroplets
Source: Chem Sci. 2026 Mar 5;17(16):8281–90. doi: 10.1039/d6sc00238b (PMC12969873; doi:10.1039/d6sc00238b)
Supplement: SC-017-D6SC00238B-s001 [file SC-017-D6SC00238B-s001.pdf]

Supplementary Information (SI) for Chemical Science.
This journal is © The Royal Society of Chemistry 2026

Supplementary Information

Non-covalent Assembly-Enabled Selectivity in Aqueous Microdroplets

Zhiheng Ma,[a] Pengju Wu,[a] Xianlong Zhou*,[a] Lingchao Cai,[a] Thomas Heine*,[b,c,d] Yu Jing*[a]

[a] Jiangsu Co-Innovation Centre of Efficient Processing and Utilization of Forest Resources, College of Chemical Engineering, Nanjing Forestry University, Nanjing 210037, China

[b] TU Dresden, Fakultät für Chemie und Lebensmittelchemie, Bergstraße 66c, 01062 Dresden, Germany

[c] Center for Advanced Systems Unerstanding (CASUS), Helmholtz-Zentrum Dresden-Rossendorf, Am Untermarkt 2, 02826 Görlitz, Germany

[d] Department of Chemistry and ibs-cnm, Yonsei University, Seodaemun-gu, Seoul 120-749, Republic of Korea

Email: xianlongzhou@njfu.edu.cn; thomas.heine@tu-dresden.de; yujing@njfu.edu.cn

Table of Contents

Methods s1

Supplemental Figures S1-S32 ....s4

Supplemental Tables S1-S3 ....s20

References s22

Methods

Chemicals and materials

5-Hydroxymethylfurfural (98%, HMF), furfural (99%, FAL), furfuryl alcohol (99%, FOL), 5-methylfurfural (98%), 5-methoxymethyl-2-furfural (97%) and FmA were purchased from Sane Chemical Technology (Shanghai) Co., Ltd (China). Chromatographic grade methanol was purchased from Shanghai Aladdin Biochemical Technology Co., Ltd (China). Milli-Q water (18.2 $M\Omega \cdot cm^{-1}$) used in all experiments was obtained from the Barnstead Easypure II system (Thermo Fisher Scientific, Waltham, MA, USA). Solutions of the chemicals with specific concentrations were prepared by dissolving them in water.

Microdroplet generation and mass spectrometry analysis

The microdroplet generating system comprises an infusion pump loaded with an aqueous substrate mixture and a precision pusher platform (**Fig. S1d**) to deliver the solution through a fused silica capillary (inner capillary). A coaxial out capillary is concentrically aligned to form an annular space for sheath gas flow. A high-pressure N_2 sheath gas supplied from a gas cylinder is delivered through a three-way connector into the annular space, generating a focused microdroplet spray (with microdroplets of 1 to 50 μm in diameter).[1] The fused silica capillary used for spraying is coated with Polymicro's standard Type 12 polymer coating. The inner diameter of its starting section is 98 μm, and the outer diameter is 191 μm. The protective sleeve for the fused silica capillary is Model T-6805, which is made of PTFE, with an outer diameter of 1/16 inch and an inner diameter of 0.2 mm.The coaxial sheath gas channel tube has an outer diameter of 1/16 inch and an inner diameter of 0.5 mm.The precision push rod platform is driven by a stepper motor and is equipped with a syringe adapter (compatible with 500 μL Hamilton syringes). It is directed toward the inlet of the mass spectrometer (MS). The distance between the spray emitter and the MS inlet is defined as the reaction distance. The percentage of the product was calculated using the peak intensities according to the formula:$\sum I(\text{product})/\sum[I(\text{product}) + I(\text{reactant})]$, where I represents the intensity of the corresponding peaks.

The solution was sprayed at a flow rate of 5-20 μL/min using a syringe pump, and the syringe propulsion pump can be tuned to control the flow rate. High-purity nitrogen was used as the nebulizing

gas at pressures of 80 to 160 psi. The inner diameter (I.D.) of the fused silica capillary used for spraying was 100 μm. Unlike electrospray ionization, no voltage was applied to the solution. The distance between the silica capillary tip and the mass spectrometer inlet defined as the reaction distance, was varied from 5 to 20 mm. The products were detected and analyzed by an LTQ-XL mass spectrometer (Thermo-Fisher, Waltham, MA). The inlet capillary temperature of the mass spectrometer was maintained at 275 °C. Collision-induced dissociation (CID) was performed for structural analysis of the products. All these experiments were performed under atmospheric pressure.

Droplet collection and GC-MS analysis

The droplet collection process is as follows: The tip of the quartz capillary tube is inserted into the collection bottle. An ice bath setup is positioned beneath the collection bottle to cool and collect the sprayed mist. A condenser reflux system is installed above the bottle opening to enhance collection efficiency. The collected droplets are subsequently analyzed using GC-MS (GCMS-QP2010 SE, SHIMADZU Co., Ltd.). The ^{1}H NMR spectra (400 MHz, $CDCl_3$) of HMF solutions with incremental concentration of HMF (1, 10, 50, and 100 μL) were recorded to monitor the chemical shift of the aldehyde proton (Ascend 400, Bruker Corporation).

Computational details

All quantum chemical calculations were performed using the Gaussian16 software package.[2] The M06-2X functional was employed in this study, which was demonstrated to be reliable in dealing with thermochemistry and non-covalent interactions.[3] The geometry of reactants, intermediates and products were obtained by fully optimization in the gas phase using the 6-31G(d) basis set because the combination of M06-2X and 6-31G(d) can well reproduce the bond length and bond angles of the aldehyde group (**Table S1**).[4-6] The SMD implicit solvation model was incorporated to account for solvent effects during the simulation of reaction energies. Frequency analyses were carried out at the same theoretical level to confirm the nature of the stationary points. To improve energy precision, single-point energy calculations were conducted on the optimized geometries using the larger 6-311+G(d,p) basis set, which includes dispersion functions to better describe electron-rich regions and polarization effects. The final Gibbs free energies (300 K,1 atm) were obtained by combining the single-point electronic energies with thermal corrections derived from frequency calculations. To

assess the influence of external electric fields on reaction kinetics, the "field" keyword was systematically utilized to simulate externally applied electric fields, with additional keywords explicitly defining the magnitude and direction of the electric field vector. The electric field strength was set within the range of -0.1 to 0.1 V/Å, consistent with the experimentally measured value at the air−liquid interface. The electric field direction was aligned along the reaction coordinate axis to maximize coupling with charge redistribution during transition state formation. The interaction region indicator (IRI) analysis of weak interactions was performed using Multiwfn. The expression is as follows:

$$\mathrm{IRI(r)}=\frac{|\nabla\rho(r)|}{[\rho(r)]^{\mathrm{a}}} \quad (1)$$

Where r denotes the position vector of atom in space, $\rho(r)$ denotes the electron density at point r and a is an adjustable exponential parameter, usually taken as 1.

The hydrogenation energy is estimated by simulating the free energy change of the initial combination of HMF with a •H radical, which is described as：

$$\Delta G = G_{HMF-H} - G_{HMF} - G_{\cdot H} \quad (2)$$

Where G_{HMF-H}, G_{HMF} and $G_{\bullet H}$ denote the Gibbs free energy of initially hydrogenated HMF, pristine HMF and hydrogen radical, respectively (**Table S2**).

AIMD simulations were performed to understand the stability of HMF oligomers at the air-water interface. The DFT optimized di-HMF was initially placed at the gas-water interface, which was constructed by placing water clusters of 146 molecules in a 25×25 ×25 $Å^3$ simulation box. AIMD simulations were performed at 300 K within the NVT ensemble. The Nosé-Hoover method was used to control simulations lasting for 40 ps with a time step of 1.0 fs.

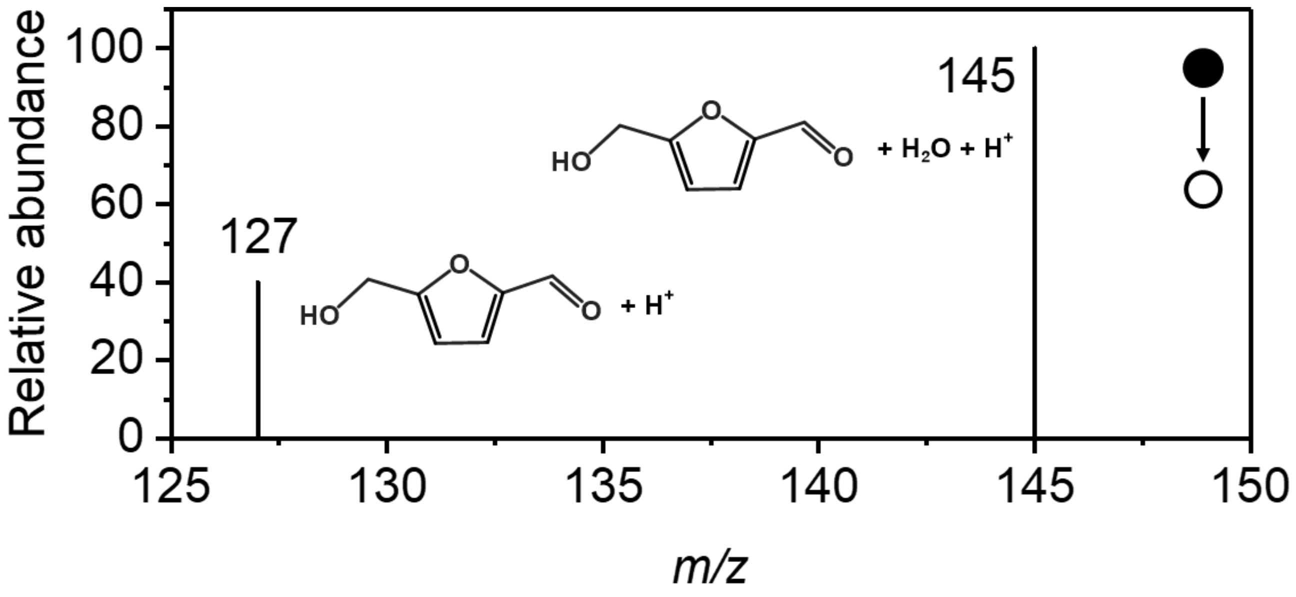

Fig. S1 MS^2 of the m/z 145 product of HMF in the positive mode.

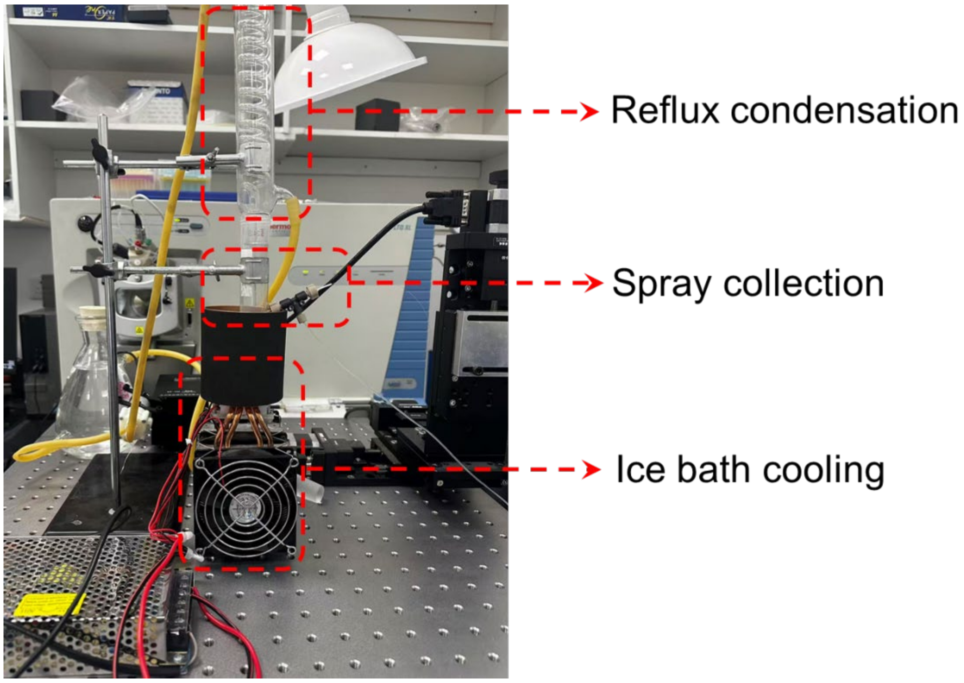

Fig. S2 Photograph of the offline microdroplets collection setup.

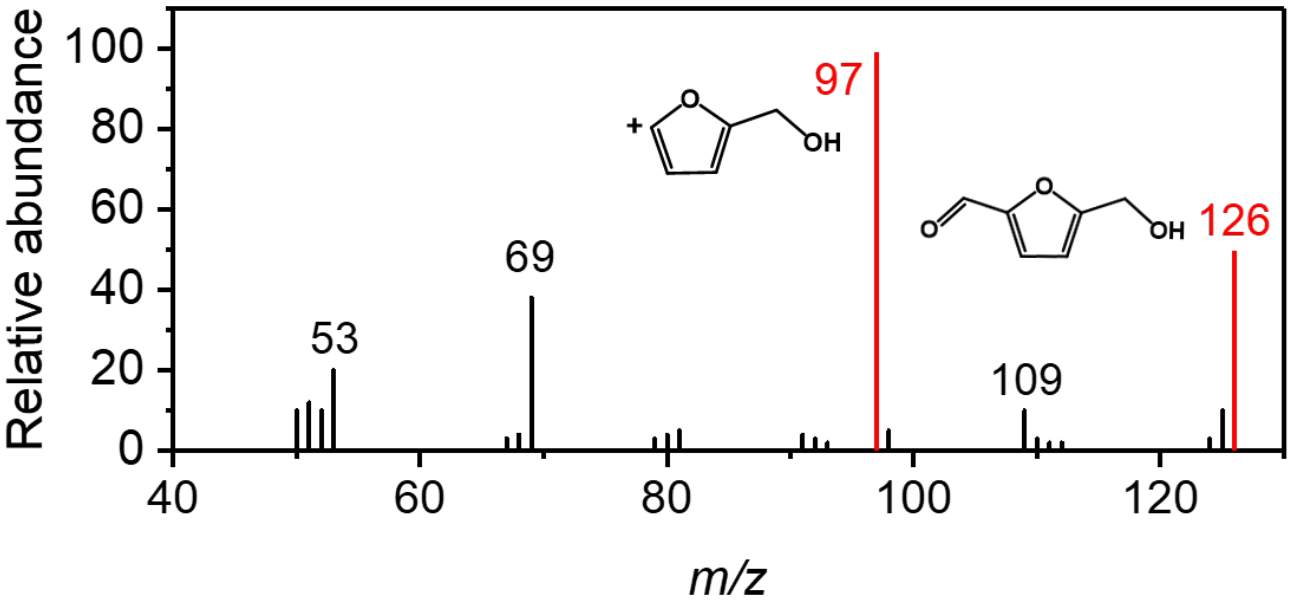

Fig. S3 GC-MS mass spectrum corresponding to the 15 min retention time.

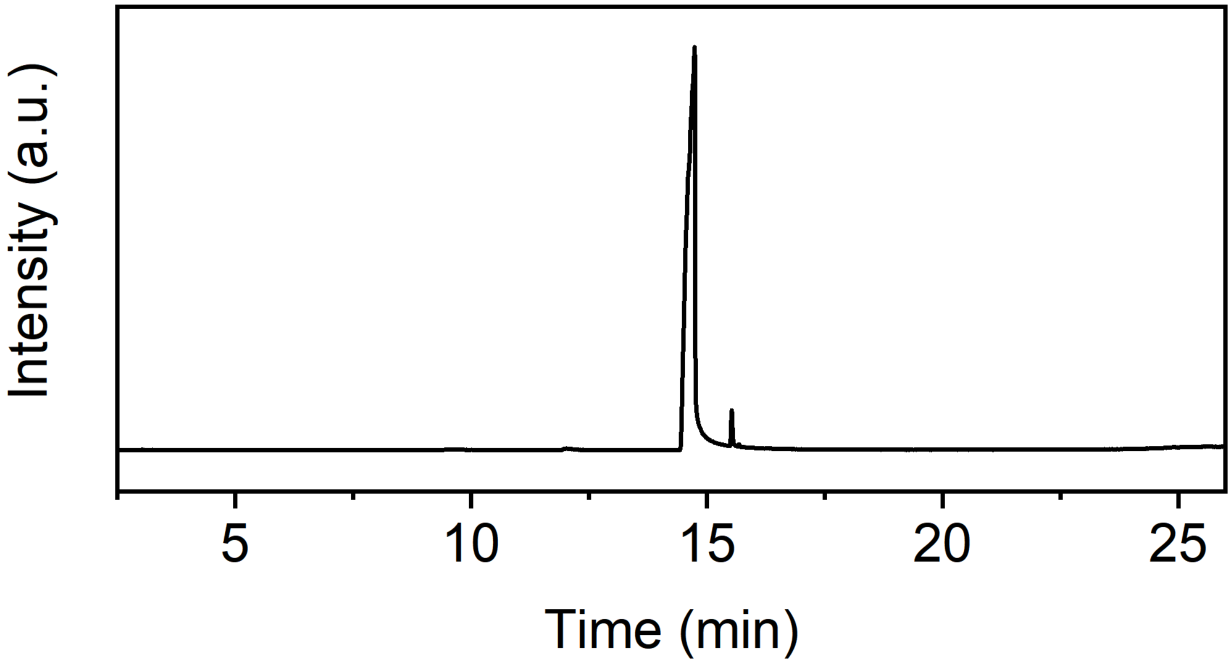

Fig. S4 GC-MS chromatogram of bulk HMF solution.

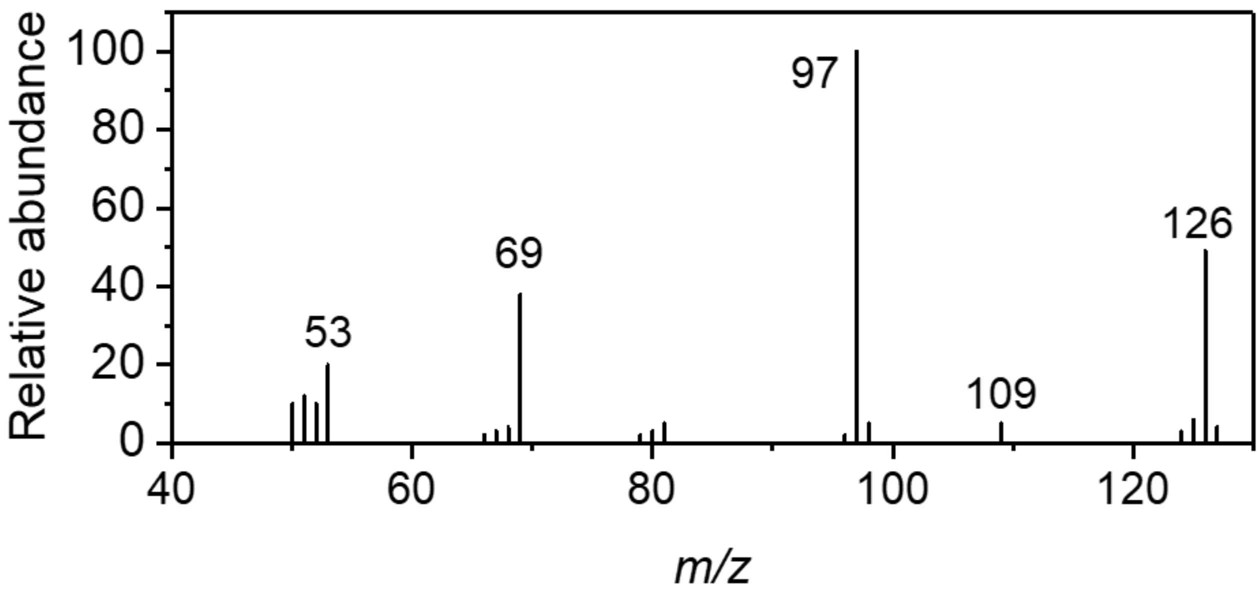

Fig. S5 GC-MS mass spectrum of bulk HMF solution analyzed at the 15 min retention time.

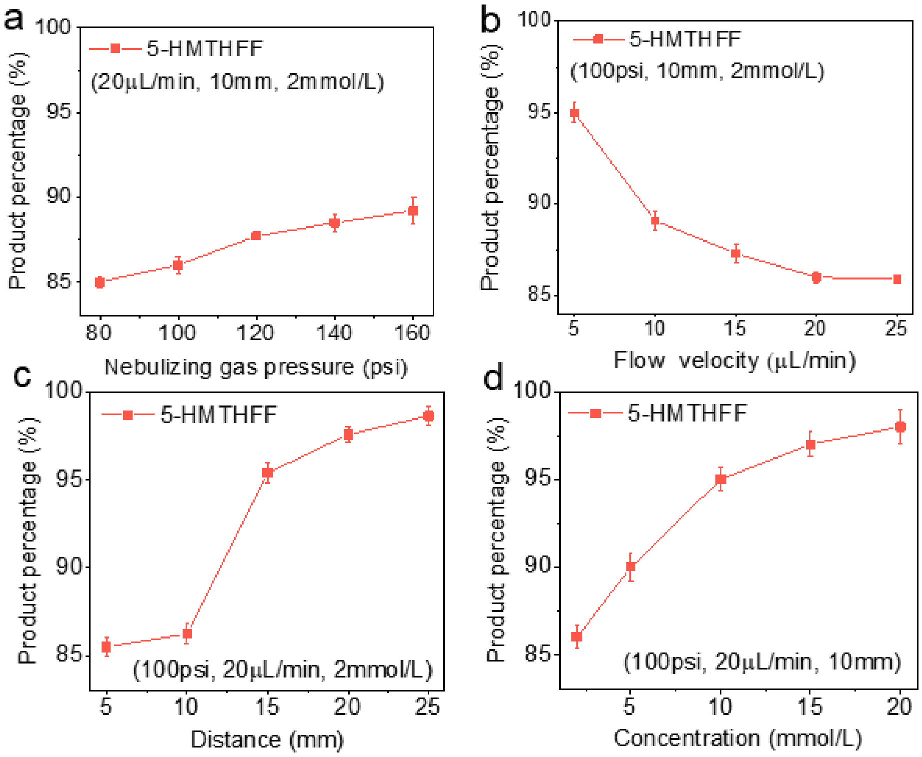

Fig. S6 Yield of 5-HMTHFF as a function of (a) gas pressure, (b) flow velocity, (c) reaction distance and (d) HMF concentration, respectively.

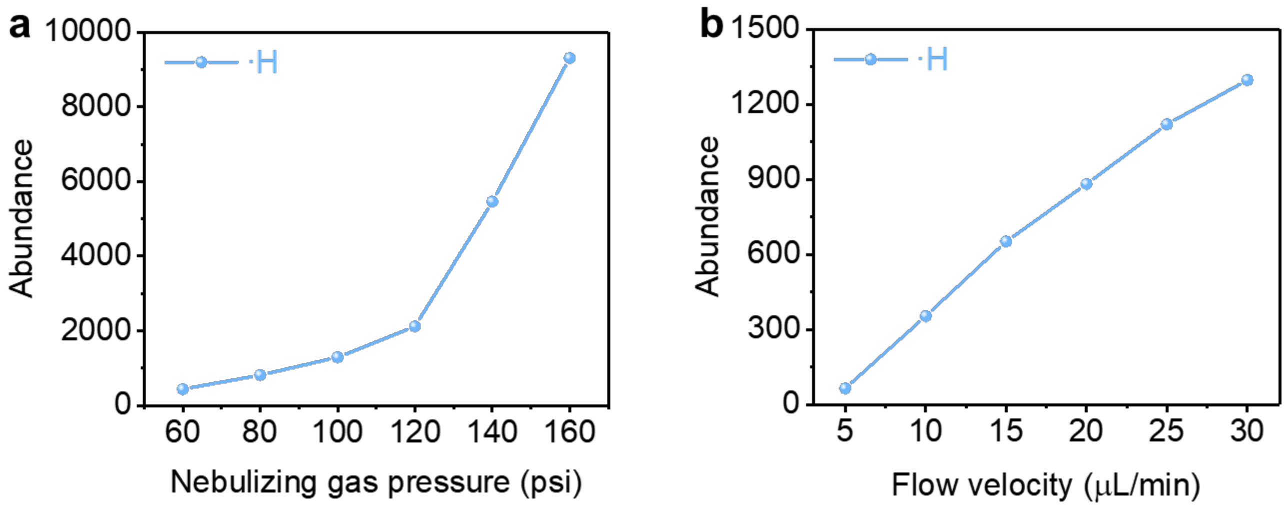

Fig. S7 Abundance of trapped •H as a function of (a) gas pressure and (b) flow velocity, respectively.

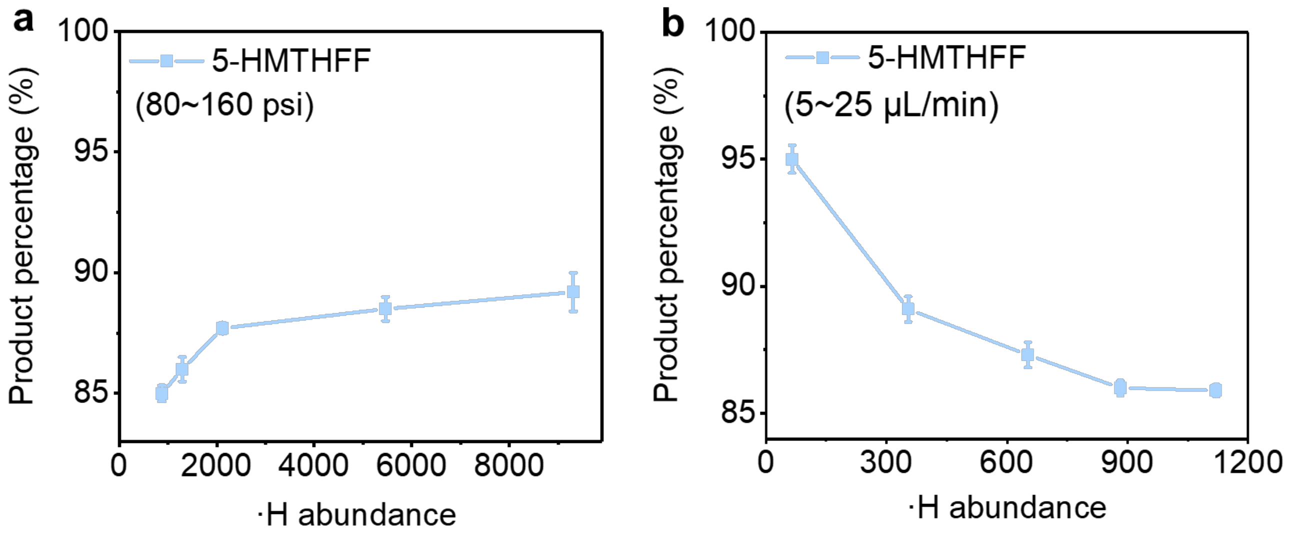

Fig. S8 Abundance dependence of 5-HMTHFF yield on the hydrogen radical concentration under (a) different gas pressures and (b) different flow rates, respectively.

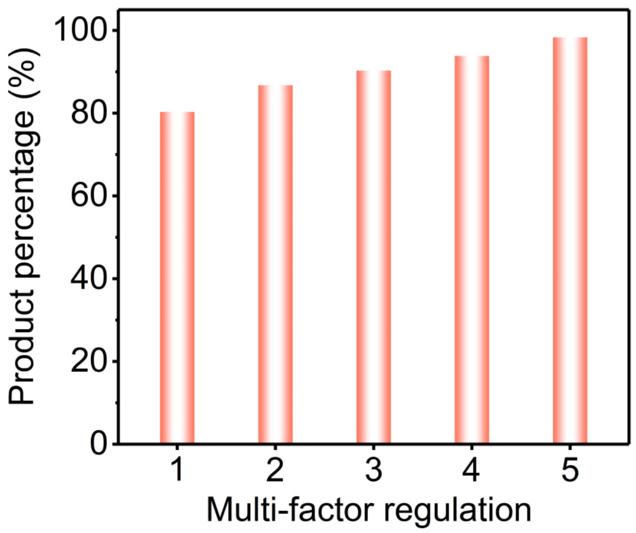

Multi-factor	1	2	3	4	5
Gas pressure (psi)	80	100	120	140	160
Distance (mm)	5	10	15	20	25
Concentration (mmol/L)	2	5	10	15	20
Flow velocity (μL/min)	5	10	15	20	25

Fig. S9 Yield of 5-HMTHFF as a function of regulation under different conditions.

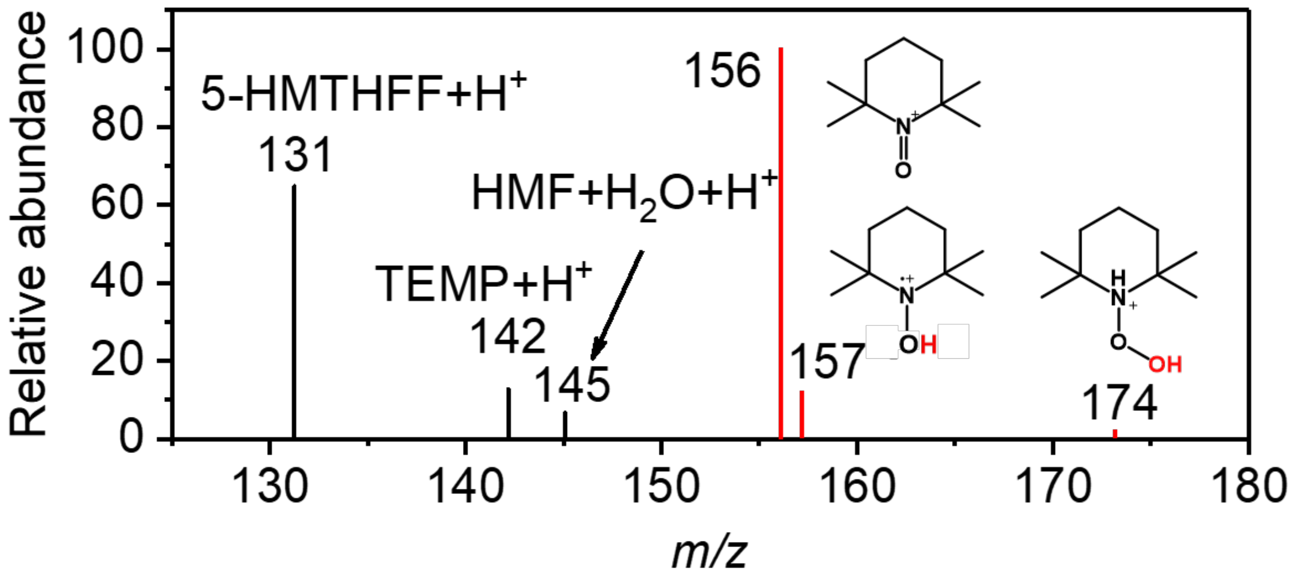

Fig. S10 Mass spectrum of HMF solution microdroplets containing TEMPO.

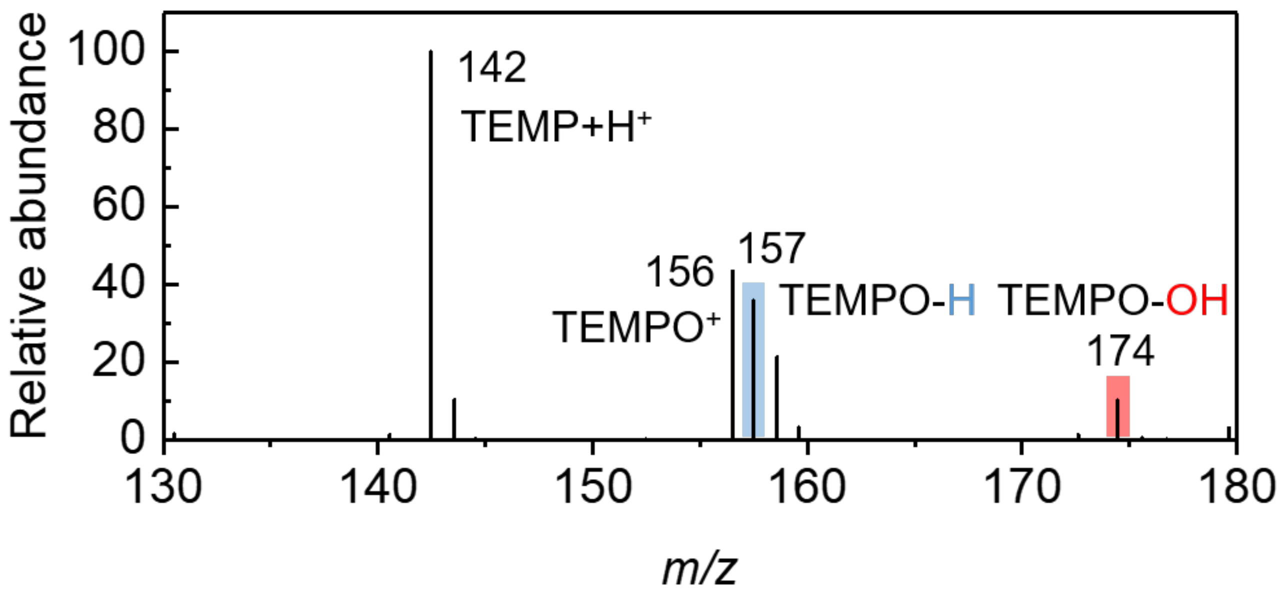

Fig. S11 Mass spectrum of H_2O microdroplets containing TEMPO.

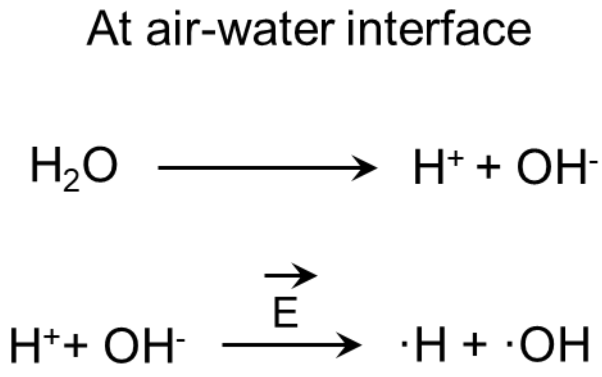

Fig. S12 Mechanism for •H generation from water at the air-water interface of microdroplets.

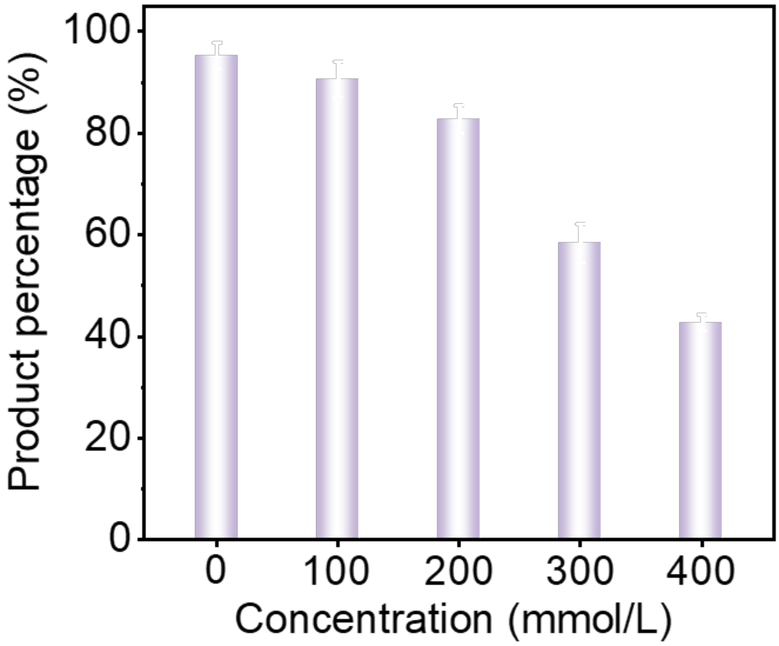

Fig. S13 Yield of 5-HMTHFF as a function of TEMPO concentration .

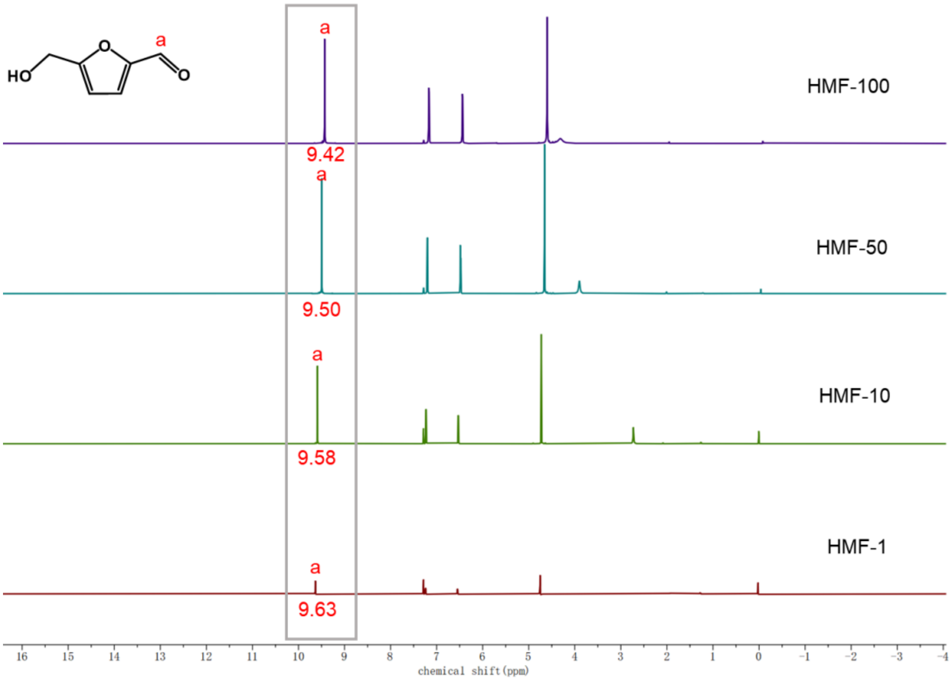

Fig. S14 ^{1}H NMR spectra of HMF with different concentrations (1, 10, 50, 100 μL in $CDCl_3$).

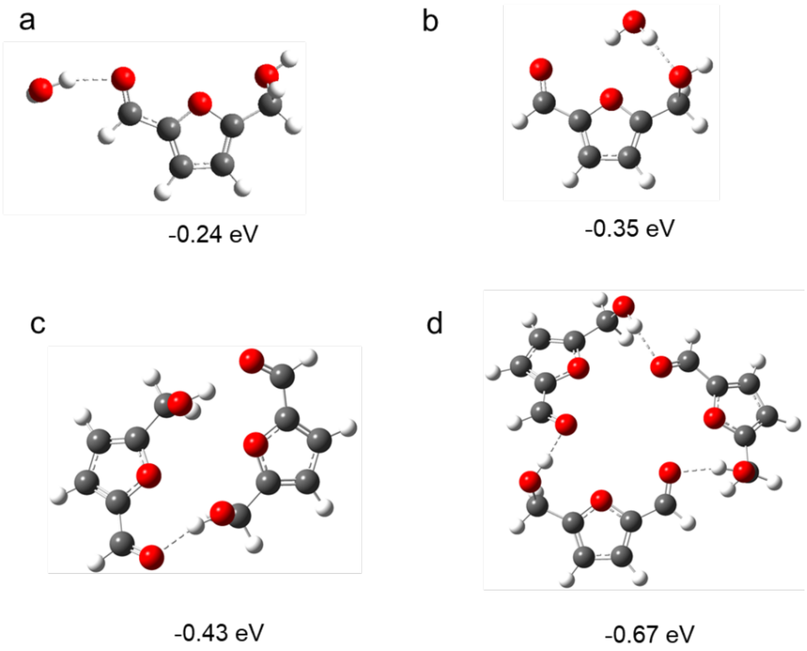

Fig. S15 The binding energy for the hydrogen bond between one water molecule and the aldehyde group (a) or the hydroxyl group (b) of HMF, and that between HMFs in the dimer (c) and trimer (d) configurations, which corresponds to-5.53, -8.07, -9.92 and -15.45 kcal/mol, respectively.

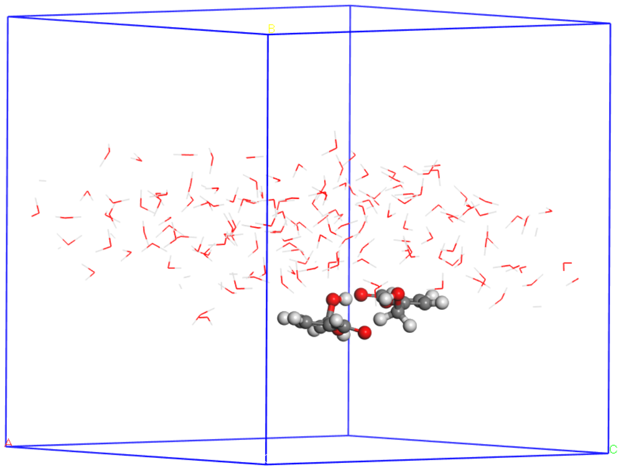

Fig. S16 Schematics for di-HMF at the air-water interface in the AIMD simulations.

According to the DFT optimized configuration of di-HMF in the explicit solvent, the length of the intermolecular hydrogen bond is about 1.85Å. We then placed the optimized di-HMF at the air-water interface and performed AIMD simulations (Fig. S8). It can be found from Fig. S9 that the hydrogen bonds fluctuate at a distance smaller than 2.0 Å. Specifically, the hydrogen bonds are mainly distributed at around 1.8 Å, demonstrating the stable configuration of di-HMF at the interface.

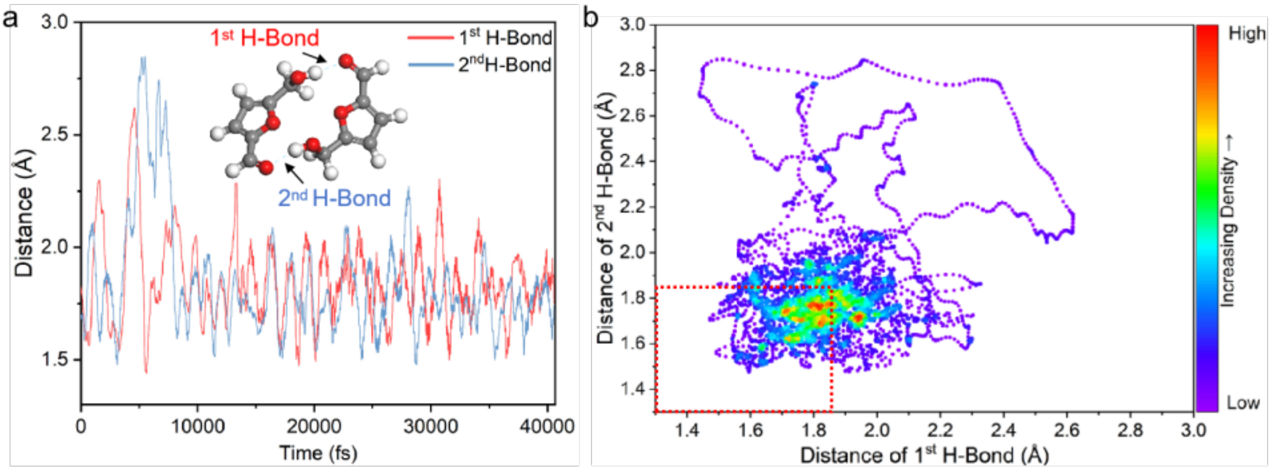

Fig. S17 (a)variation of the intermolecular hydrogen bond length of di-HMF as function of the simulation time; (b) Heat map for the distribution of the hydrogen bond distance of di-HMF during AIMD simulations.

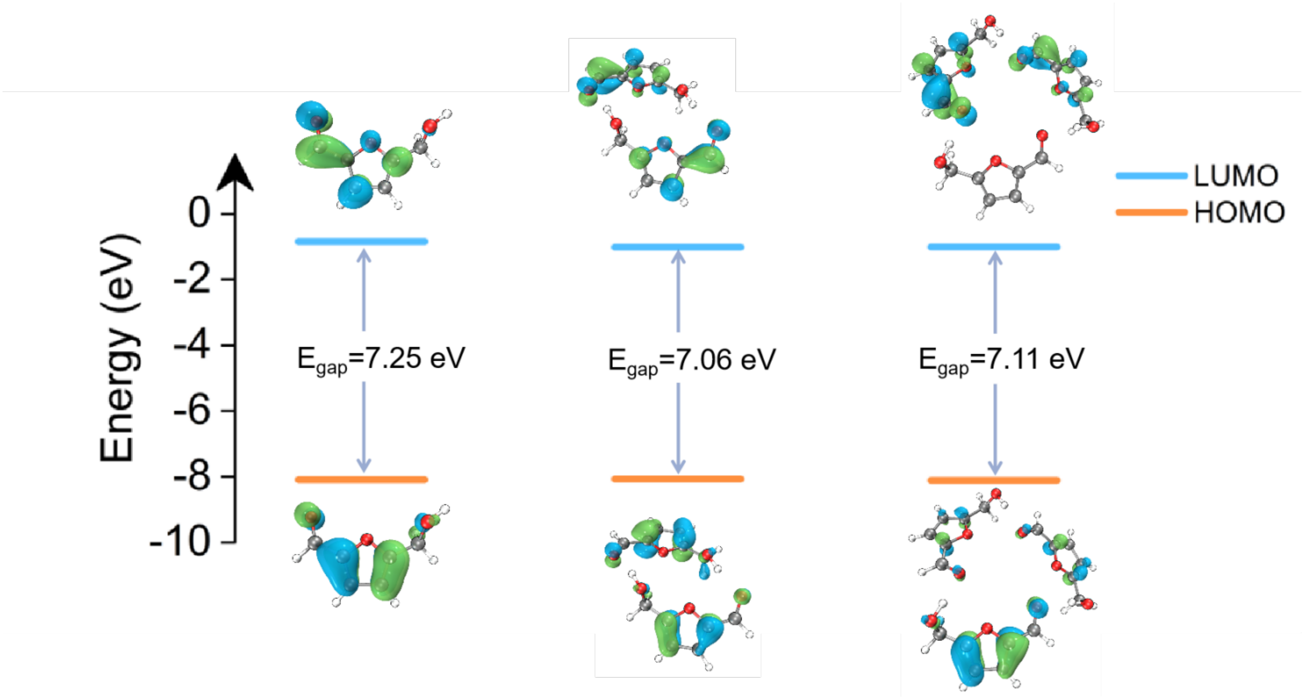

Fig. S18 The calculated frontier orbitals and HOMO-LUMO energy gaps of HMF monomer, dimer and trimer.

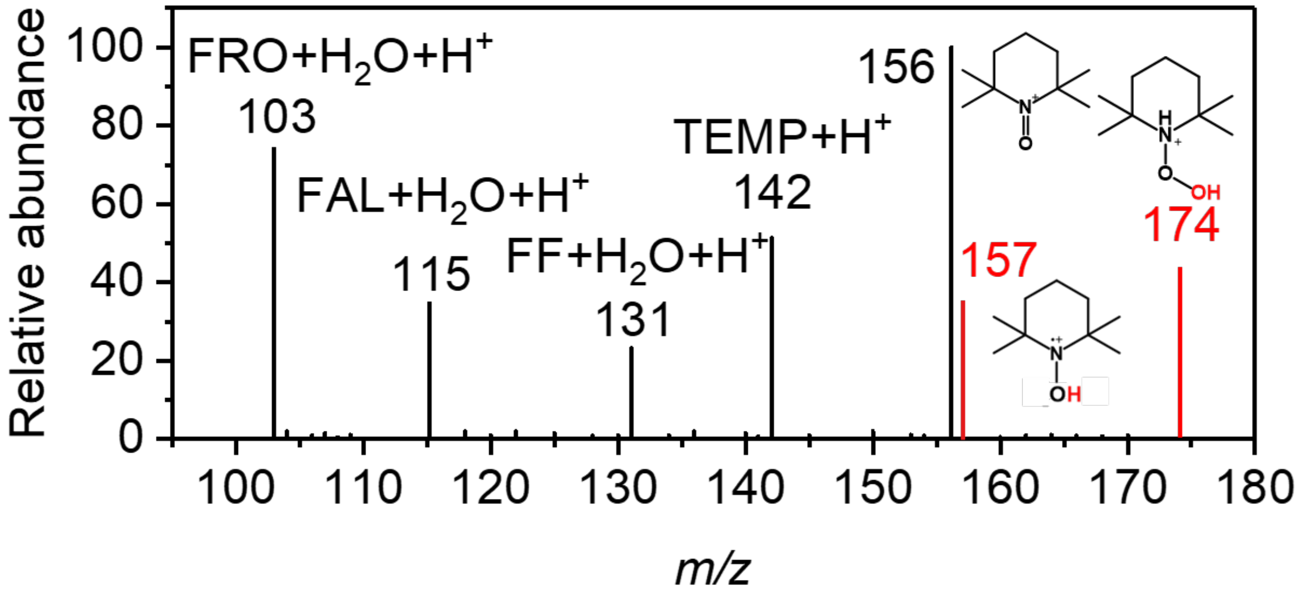

Fig. S19 Mass spectrum for the sprayed FAL solution microdroplets containing TEMPO.

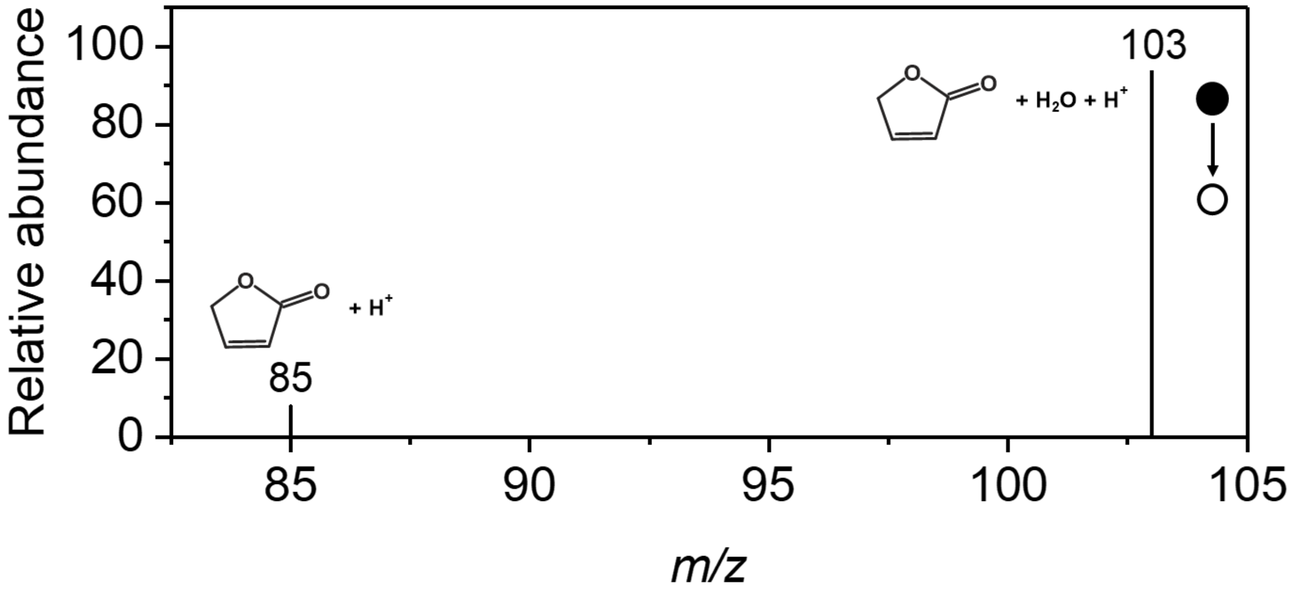

Fig. S20 MS^2 of the adduct observed at m/z 103 in positive mode when spraying the FAL solution.

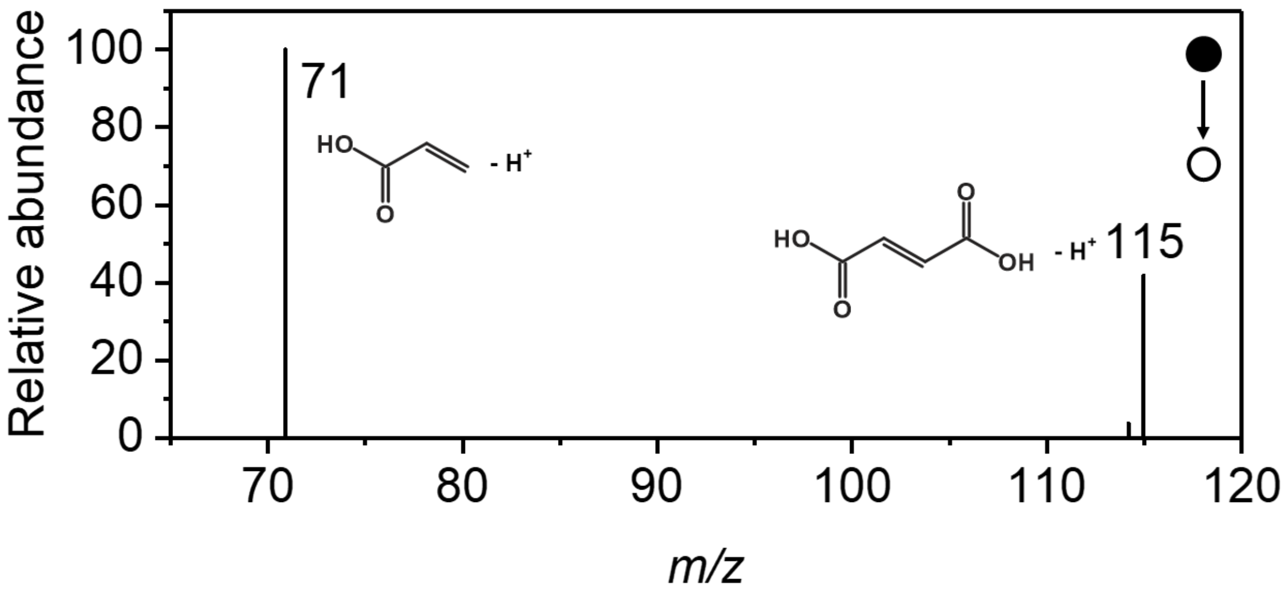

Fig. S21 MS^2 of the m/z 115 product in negative mode when spraying the FAL solution.

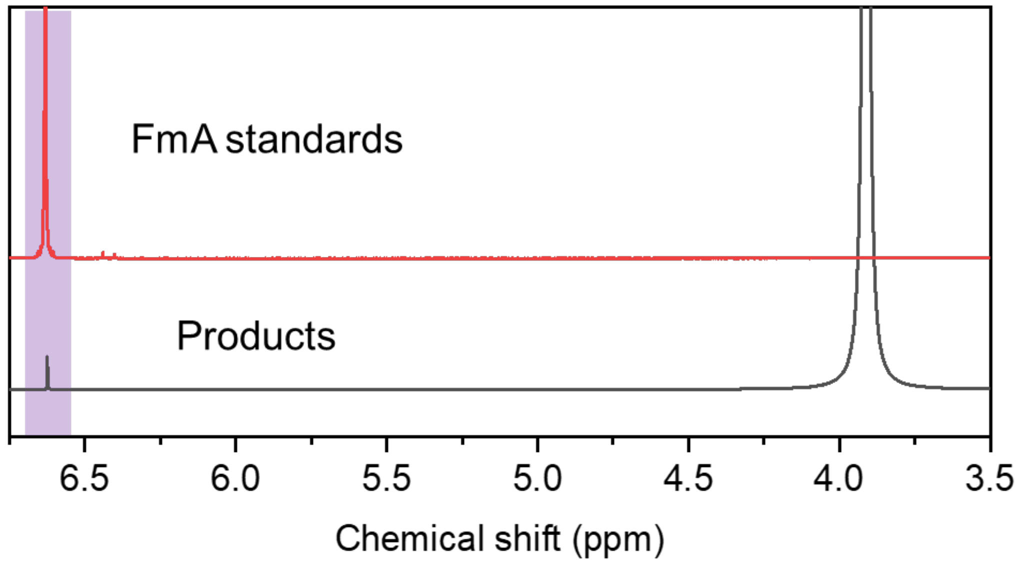

Fig. S22 ^{1}H NMR spectra of collected FAL spray microdroplets (black) in comparison with that of the authentic FmA standard sample (red).

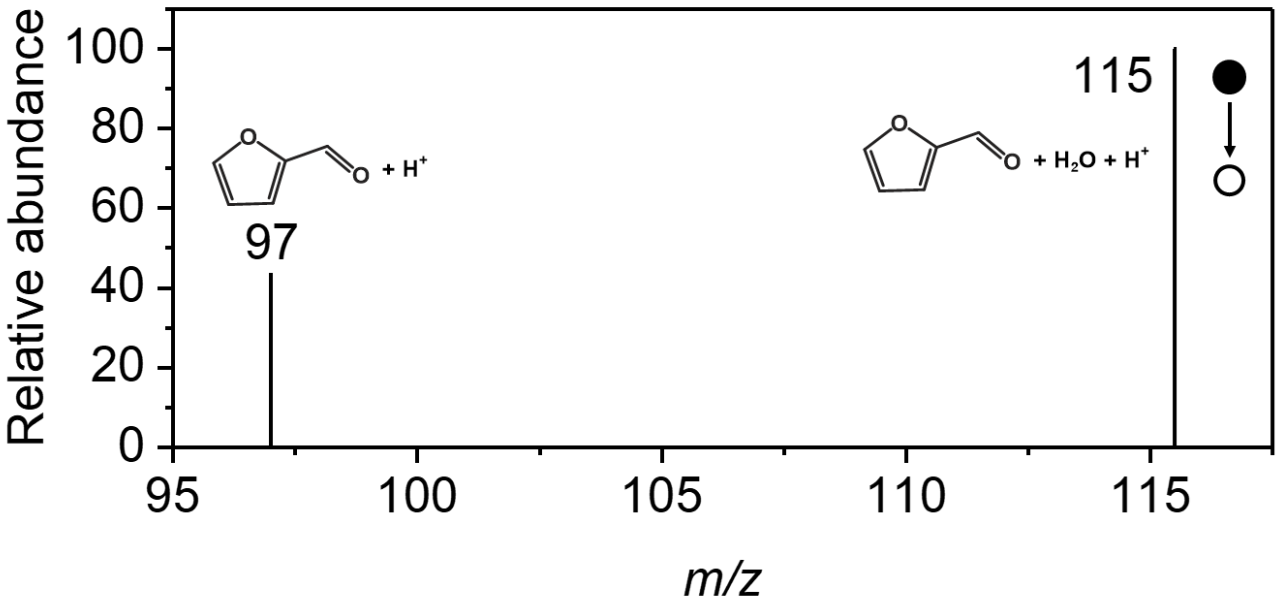

Fig. S23 MS^2 of the m/z 115 product in positive mode when spraying the FAL solution.

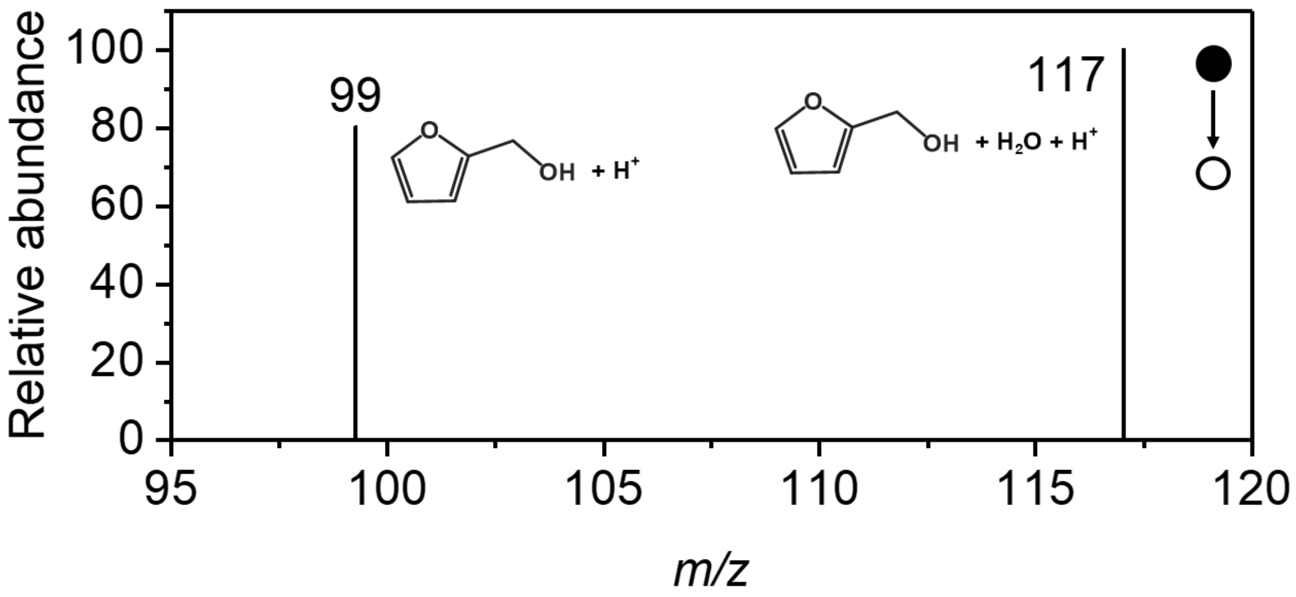

Fig. S24 MS^2 of the m/z 117 product in positive mode of when spraying the FAL solution.

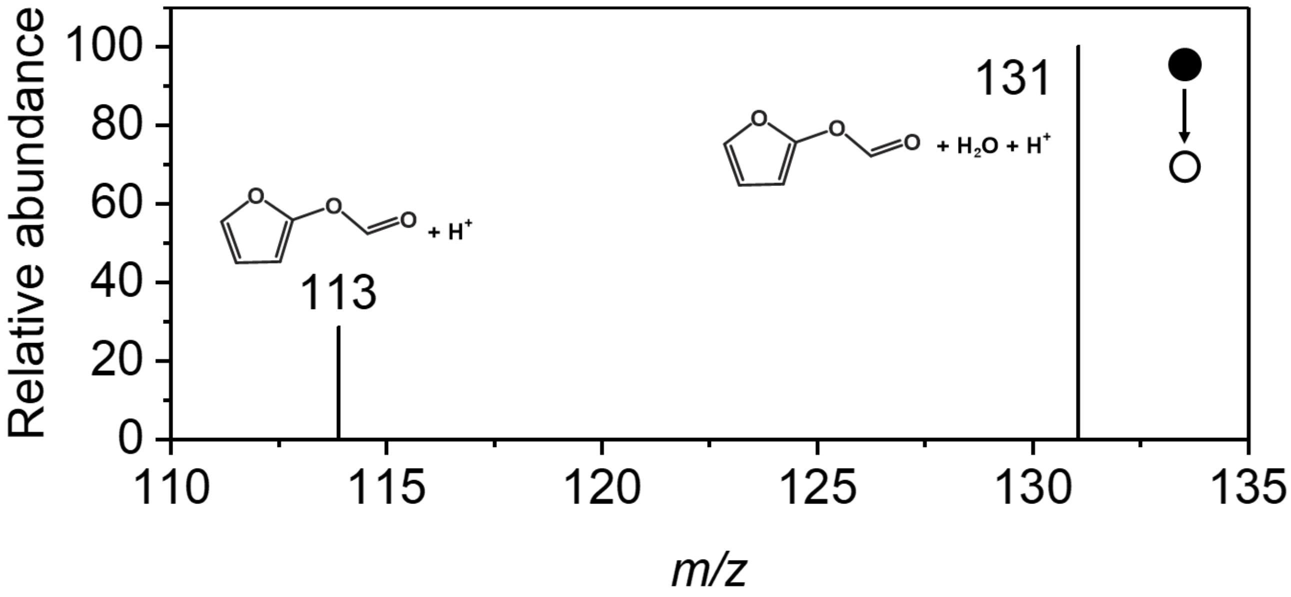

Fig. S25 MS^2 of the m/z 131 product in positive mode when spraying the FAL solution.

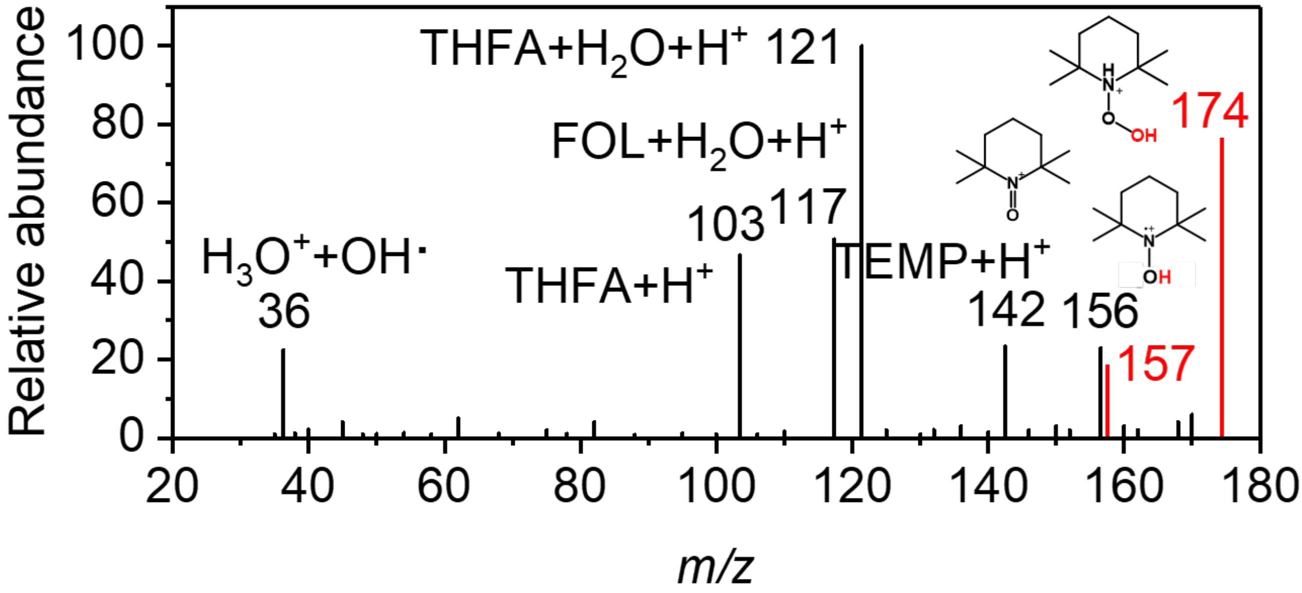

Fig. S26 Mass spectra when spraying FOL solution containing TEMPO

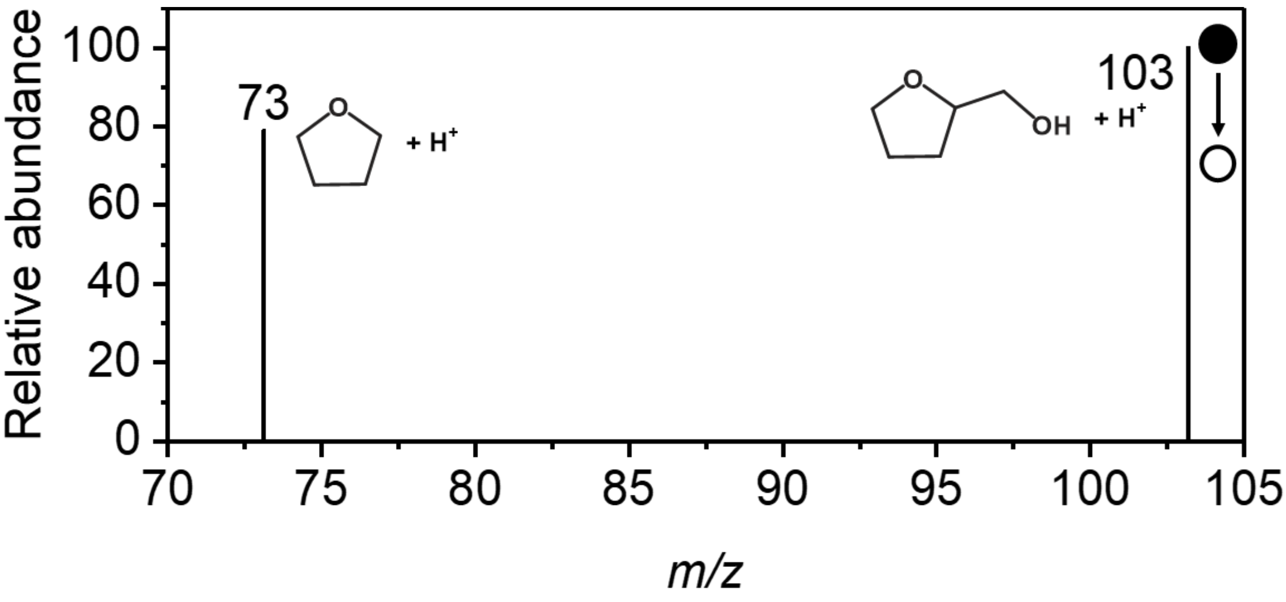

Fig. S27 MS^2 of the m/z 103 product in positive mode when spraying the FOL solution.

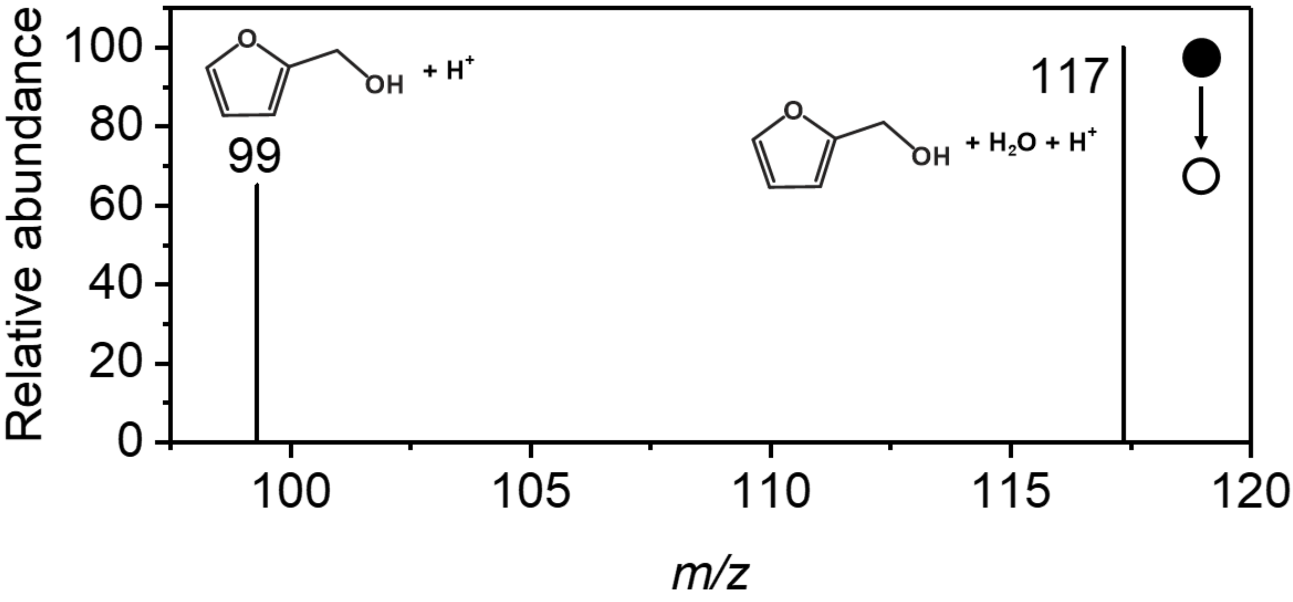

Fig. S28 MS^2 of the m/z 117 product in positive mode when spraying the FOL solution.

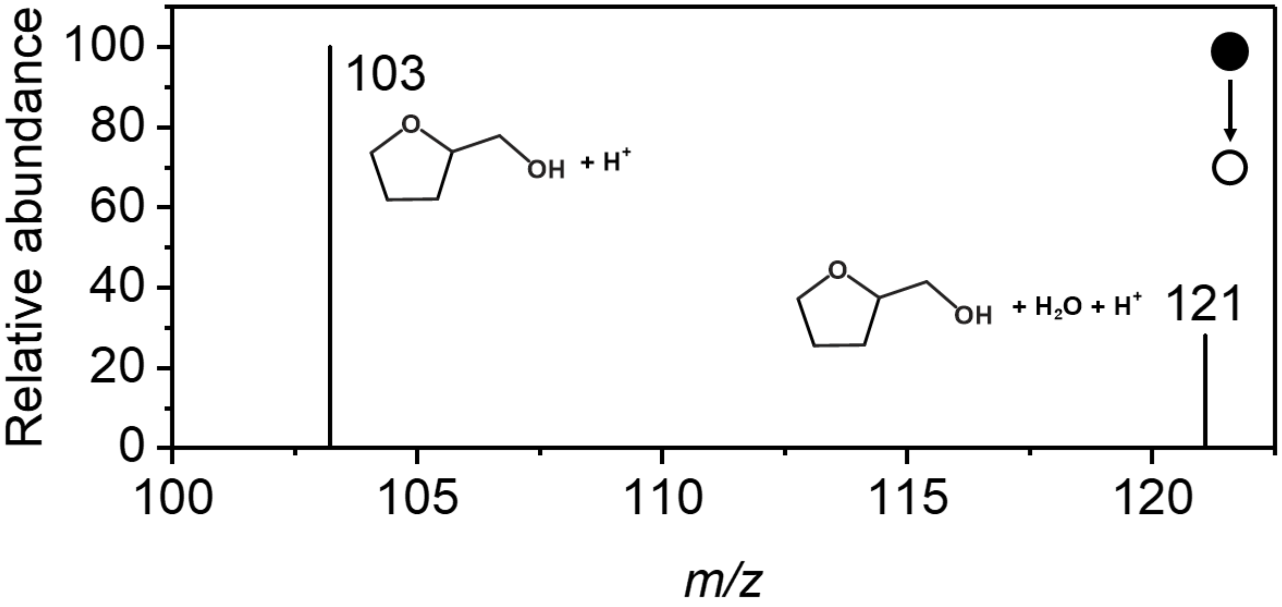

Fig. S29 MS^2 of the m/z 121 product in positive mode when spraying the FOL solution.

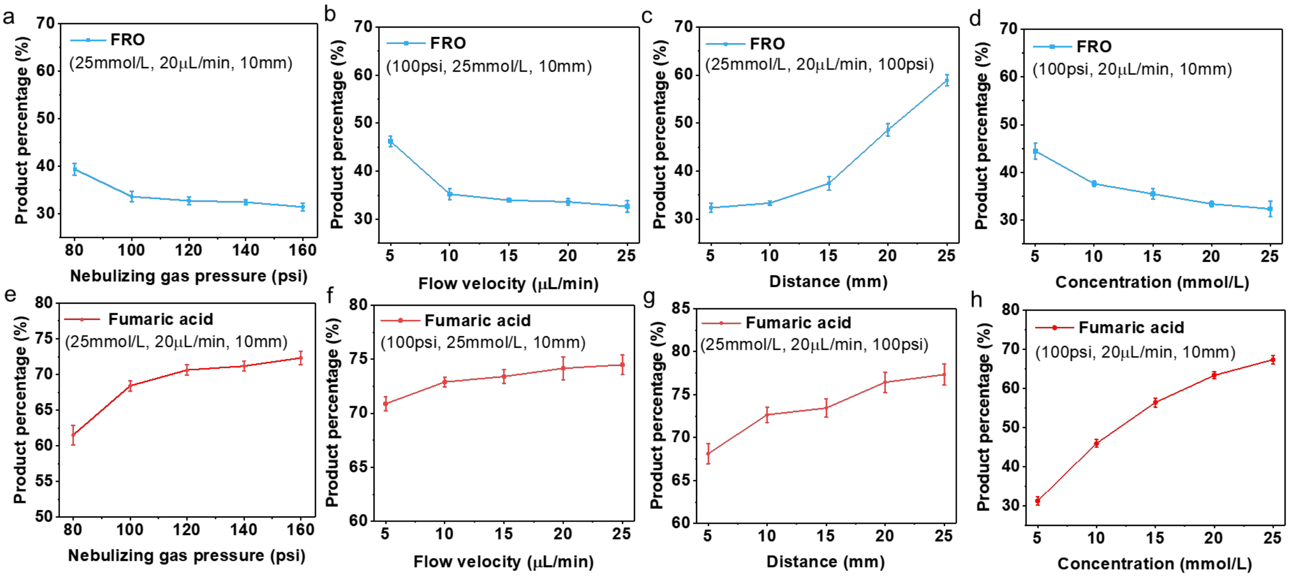

Fig. S30 Product percentage of FRO (a-d) and FuA (e-h) generated by spraying FAL solution as a function of the sheath gas pressure, flow rate of the reactant, reaction distance and concentration of the reactant, respectively.

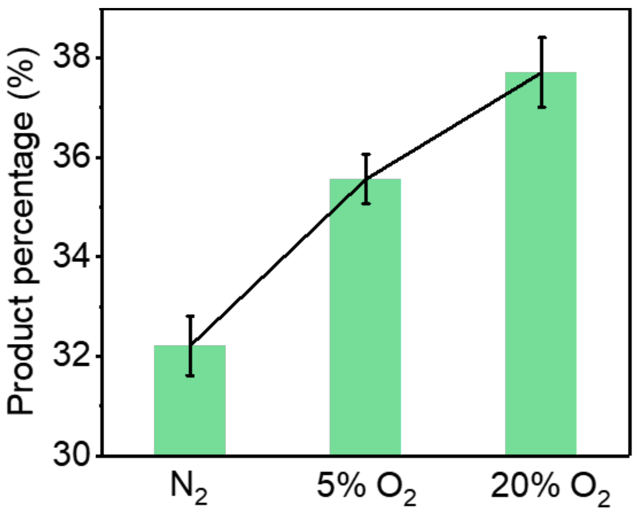

Fig. S31 Effect of different sheath gases, including pure N_2 gas, N_2 gas containing 5% O_2 and N_2 gas containing 20% O_2, on the product percentage of FRO generated by FAL solution (25 mmol/L) spraying at 100 psi with the reaction distance of 10 mm.

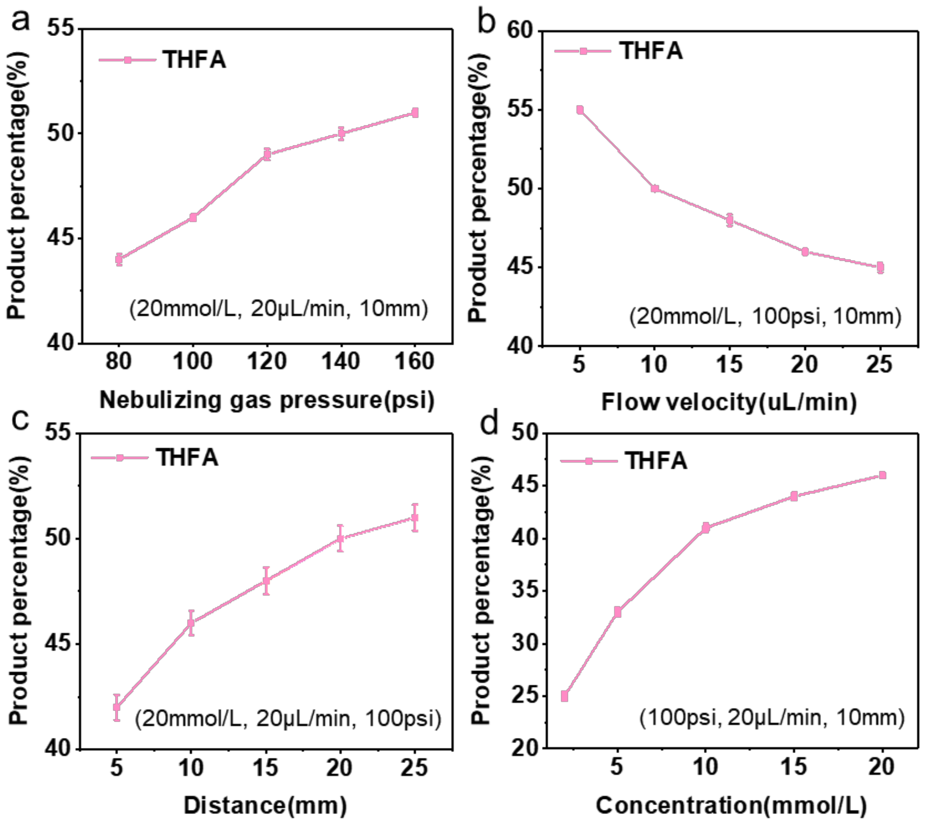

Fig. S32 Product percentage of tetrahydrofurfuryl alcohol (THFA) generated by spraying FOL solution as a function of sheath gas pressure, flow rate of the reactant, reaction distance and concentration of the reacta.

Table S1 Calculated bond length (rC-C and rC=O in Å) and bond angle (wC-C-O in Å) of the aldehyde group of HMF in aqueous phase using M062X or B3LYP functional with the combination of different basis sets, in comparison with the experimental observed values.

Functional	Basis set	rC-C	rC=O	wC-C-O
M062X	6-31+g(d)	1.445	1.226	124.08
**M062X**	**6-31+g(d,p)**	**1.445**	**1.226**	**124.01**
M062X	6-311+g(d)	1.444	1.219	124.32
M062X	6-311+g(d,p)	1.444	1.219	124.18
B3LYP	6-31+g(d)	1.434	1.238	125.09
B3LYP	6-31+g(d,p)	1.434	1.238	125.02
B3LYP	6-311+g(d)	1.433	1.231	125.28
B3LYP	6-311+g(d,p)	1.434	1.231	125.15
Expt.		1.453	1.212	122.7

Table S2 The H binding energy (in eV) at different sites of HMF molecule.

HMF	Furfural-C_4	Furfural-C_3	Furfural-C_2	Furfural-C_1	Aldehyde-O
monomer	-1.76	-1.16	-1.31	-1.07	-1.64
dimer	-2.10	-1.11	-1.81	-1.48	-1.87
trimer	-2.10	-1.12	-1.81	-1.45	-1.88

Table S3 Comparison of 5-HMTHFF formation from HMF under different reaction conditions. The product yield reported in this work was estimated based on MS analysis, according to equation:∑I(product)/∑[I(product) + I(reactant)], where I represents the intensity of the corresponding peaks.

**Method**	**Catalyst**	**Catalyst mass (mg)**	**Temperature (°C)**	**Pressure (MPa)**	**Time**	**Gas**	**Product**	**Yeild (%)**
Thermal catalysis	Pd/LDH-MgAl-NO_3	10	30	1	4 h	H_2	5-HMTHFF<br>BHMF<br>BHMTHF	81.4
Thermal catalysis	Pd-MgO-Al_2O_3	100	40	4	2 h	H_2	5-HMTHFF<br>BHMF<br>BHMTHF	82.4
Thermal catalysis	Pd/ZrO	20	ambient temperature	1	4 h	H_2	5-HMTHFF<br>BHMTHF	92.3
Thermal catalysis	Pd@NSi @Al_2O_3	100	5	1	4 h	H_2	5-HMTHFF<br>BHMF<br>BHMTHF	83.5
**Micro-droplet**	/	/	ambient temperature	/	**ms**	$\mathbf{N_2}$	**5-HMTHFF**	**98.3**

Reference

1 M. T. Dulay, C. F. Chamberlayne, R. N. Zare, *Anal. Chem.*, 2022, **94**, 3762-3766.

2 Frisch MJ, Trucks GW, Schlegel HB, Scuseria GE, Robb MA, Cheeseman JR, et al. Gaussian 16 Rev. B.01. Wallingford, CT2016.

3 Y. Zhao, D. G. Truhlar, *Theor. Chem. Acc.*, 2008, **120**, 215-241.

4 T. Clark, J. Chandrasekhar, G. W. Spitznagel, P. V. R. Schleyer, *J. Comput. Chem.*, 1983, **4**, 294-301.

5 B. P. Pritchard, D. Altarawy, B. Didier, T. D. Gibson, T. L. Windus, *J. Chem. Inf. Model.*, 2019, **59**, 4814-4820.

6 K. L. Schuchardt, B. T. Didier, T. Elsethagen, L. Sun, V. Gurumoorthi, J. Chase, J. Li, T. L. Windus, *J. Chem. Inf. Model.*, 2007, **47**, 1045-1052.
